# Variations of structural–functional coupling in post-traumatic stress disorder are associated with underlying molecular and transcriptional features

**DOI:** 10.1017/S0033291726104838

**Published:** 2026-06-18

**Authors:** Yujie Song, Bin Huang, Jiayu Wu, Jinping Lin, Kaiqi Xin, Bo Gao, Graham J. Kemp, Bin Guo, Qiyong Gong

**Affiliations:** 1 Xiamen Key Lab of Psychoradiology and Neuromodulation, Department of Radiology, West China Hospital, West China Xiamen Hospital, Sichuan University, Xiamen, China; 2 Department of Radiology, Huaxi MR Research Center (HMRRC), Institute of Radiology and Medical Imaging, Psychoradiology Key Laboratory of Sichuan Province, West China Hospital of Sichuan University, Chengdu, China; 3 Research Unit of Psychoradiology, Chinese Academy of Medical Sciences, Chengdu, China; 4 Department of Radiology, Peking Union Medical College Hospital, Chinese Academy of Medical Sciences and Peking Union Medical College, Beijing, China; 5 Department of Radiology, Affiliated Hospital of Guizhou Medical University, Guiyang, China; 6 Mental Health Center, West China Hospital, West China Xiamen Hospital, Sichuan University, Xiamen, China; 7 Liverpool Magnetic Resonance Imaging Centre (LiMRIC) and Institute of Life Course and Medical Sciences, University of Liverpool, Liverpool, United Kingdom; 8 Department of Big Data, Clinical Oncology School of Fujian Medical University, Fujian Cancer Hospital, Fuzhou, China

**Keywords:** magnetic resonance imaging, post-traumatic stress disorder, psychoradiology, structural–functional coupling

## Abstract

**Background:**

Post-traumatic stress disorder (PTSD) is a growing health problem whose neurobiology remains incompletely understood. Neuroimaging is useful in probing PTSD-related brain dysfunction, and techniques continue to evolve. Structural–functional coupling (SFC) offers a novel integrated perspective on PTSD neurobiology. We sought to define unique SFC alterations in PTSD and explore their associations with clinical symptoms, brain molecular architecture, and gene expression.

**Methods:**

We studied 61 PTSD patients and 62 trauma-exposed non-PTSD controls (TENC) recruited from earthquake survivors. We compared SFC constructed from multimodal MRI data by an eigendecomposition method between the two groups. We explored the spatial correlation of SFC with molecular maps, used partial least squares (PLS) regression to associate them with Allen Human Brain Atlas gene data, and conducted enrichment analysis on the identified genes.

**Results:**

PTSD patients showed significant regional SFC alterations in multiple regions: lower SFC in PTSD versus TENC in the default mode network (DMN), frontoparietal network (FPN), dorsal attention network, sensorimotor network, visual network, and thalamus, and higher SFC in PTSD versus TENC in the DMN, FPN, and ventral attention network. Some changes were correlated with clinical symptom severity. In both groups, the spatial distribution of SFC was similarly correlated with molecular architectures. The second component of the PLS regression genes were linked to PTSD-specific SFC variations, enriched mainly in molecular functions and pathways related to synapses and neurotransmitter signaling.

**Conclusions:**

This study yields new insights into PTSD pathophysiology by connecting macroscale SFC changes with their microscale molecular and transcriptional basis.

## Introduction

Post-traumatic stress disorder (PTSD) is a severe psychological disorder arising typically following exposure to traumatic events. Its core features are specific traumatic event-related recurrent experiences of memories, avoidance of trauma-related cues, negative alterations in cognition and mood, and hypervigilance (American Psychiatric Association, 2022). The lifetime prevalence of PTSD is ~6% worldwide, higher following severe traumas such as violent conflicts or sexual assaults (Koenen et al., 2017). With the increasing global incidence of traumatic events, including the COVID-19 pandemic, social unrest, and climate change, the burden of PTSD in the civilian population has risen significantly (Schincariol et al., 2024). Despite its profound public health impact, the neurobiological mechanisms underlying PTSD remain incompletely understood.

Neuroimaging studies have helped throw light on PTSD-related brain alterations. Functional magnetic resonance imaging (fMRI) studies probing brain network topology in PTSD have shown a shift from a random or regular configuration to more small-world network properties, with increased centrality in the default mode network (DMN) and the salience network (Lei et al., 2015), and abnormal connectivity patterns both within and between networks (Kearney & Lanius, 2024; Zhang et al., 2015; Zhu et al., 2019). Structural studies using diffusion tensor imaging (DTI) in PTSD have shown white matter microstructural anomalies such as higher white matter integrity in the inferior fronto-occipital fasciculus and inferior temporal gyrus (Ju et al., 2020), and lower fractional anisotropy in the uncinate fasciculus, cingulum bundle (O’Doherty et al., 2018), and tapetum region of the corpus callosum (Dennis et al., 2021). These findings suggest that PTSD neuropathology involves widespread neural network disruptions across regions and modalities, rather than focal variations (Akiki, Averill, & Abdallah, 2017; Zhang et al., 2025). However, single-modality techniques, structural or functional, have limited ability to characterize the interplay between brain structure and function (Mount & Monje, 2017). Studies to date exploring multimodal approaches to characterizing and assessing PTSD (Abdallah et al., 2017; Nkrumah et al., 2024; Zhang et al., 2016; Zhu et al., 2021) have not integrated multimodal information to define the patterns of structure–function coupling (SFC) needed for a holistic understanding of brain information processing.

SFC analysis is a powerful tool for multimodal integration (Calhoun & Sui, 2016) which can quantify how structural connectivity supports coordinated neural activity fluctuations, providing a coherent framework for describing multiscale brain reorganization (Baum et al., 2020). It can be implemented in three main ways: the correlational, harmonic analysis, and modeling approaches (Fotiadis et al., 2024). The stability of this integrated architecture crucially underpins the organization and coordination of brain function, and perturbations in SFC have been identified in neurological and psychiatric disorders such as epilepsy (Zhang et al., 2011), Parkinson’s disease (Zarkali et al., 2021), bipolar disorder (Collin et al., 2017), and schizophrenia (Jiang et al., 2021). SFC is also related to variations in cognitive flexibility (Medaglia et al., 2018) and general intelligence (Feng et al., 2024). SFC is therefore a promising approach to identifying unique brain abnormalities in PTSD.

To understand the pathophysiology of SFC abnormalities, we must relate macroscopic imaging findings to microscopic biological features. The biological rationale for this multilevel integration is compelling: genes play a major role in shaping network connections (Arnatkeviciute et al., 2021), as do the complex neurotransmitter interactions of diverse neural cell types; we can therefore expect all these to influence the regional distribution of SFC (Allen & Lyons, 2018; Wang, 2020; Zilles & Palomero-Gallagher, 2017). Previous studies in PTSD have confirmed its genetic susceptibility (Duncan et al., 2018), and linked its pathophysiology to mitochondrial dysfunction (Lushchak, Strilbytska, Koliada, & Storey, 2023) and neurotransmitter dysregulation (Traina & Tuszynski, 2023). However, there remains a critical gap in our understanding of the relationship between structural–functional network alterations in PTSD and regional molecular architecture and gene expression profiles.

**Figure 1.**
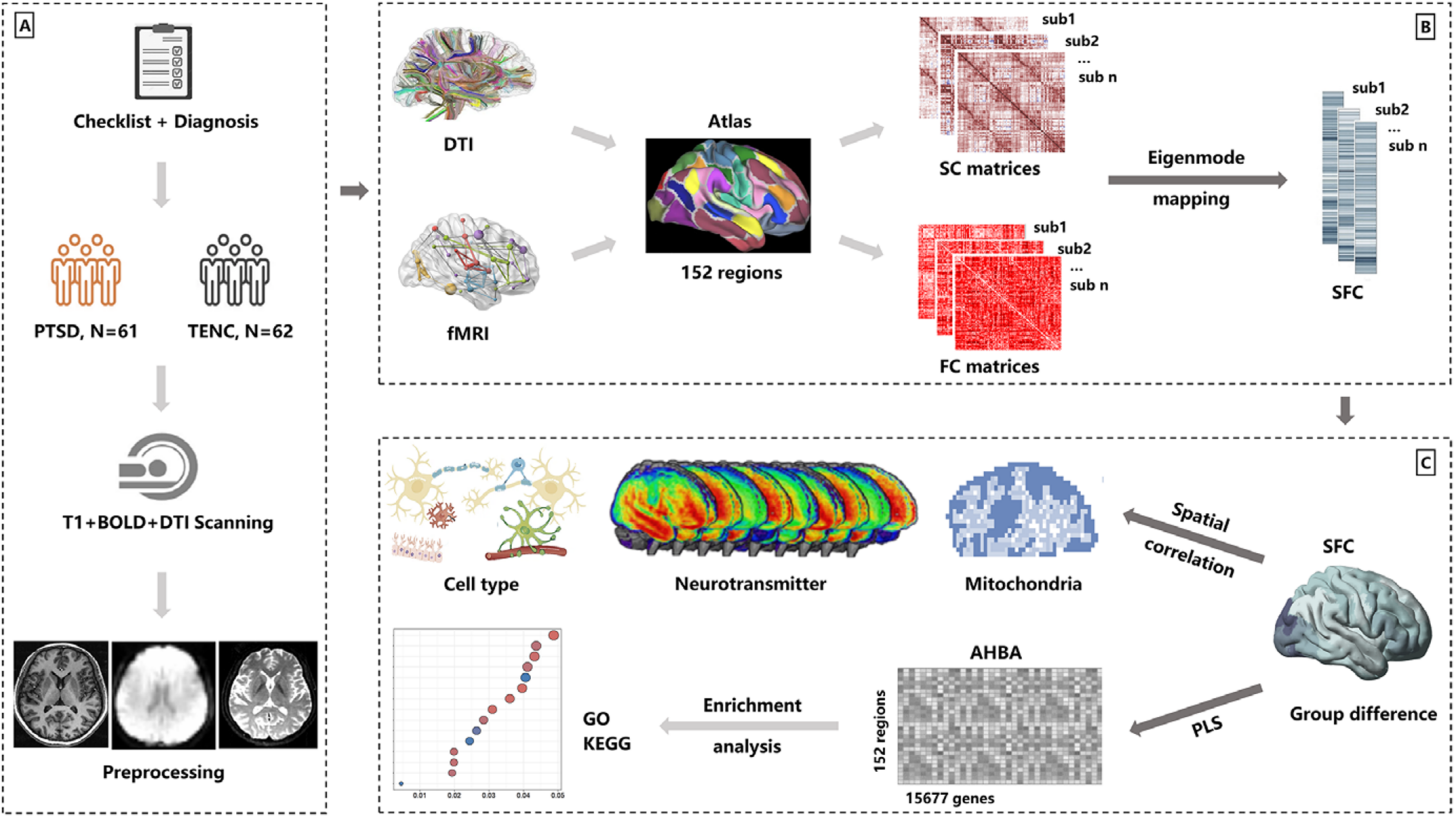
Study workflow. (a) *Participant and scanning workflow*: participants enrolled by survey had checklist assessment and clinical diagnosis; eligible subjects, classified as PTSD patients and trauma-exposed non-PTSD controls (TENC), went on to multimodal MRI scanning; MRI data preprocessing used containerized pipelines. (b) *Connectivity and structure–function coupling (SFC) analysis*: after 152-region parcellation, structural connectivity (SC) and functional connectivity (FC) matrices were constructed for each subject (sub 1, 2…n) from diffusion tensor imaging (DTI) and functional MRI (fMRI) data, respectively; then SFC was calculated by an eigenmode-mapping approach. (c) *SFC annotation and analysis*: various neurotransmitter, cell-type, and mitochondria maps were used to explore the spatial biological correlations of SFC; with gene lists from Allen Human Brain Atlas (AHBA), partial least squares regression (PLS) was used to identify SFC-related genes; enrichment analysis with Gene Ontology (GO), and Kyoto Encyclopedia of Genes and Genomes (KEGG) was used to identify relevant pathways. Figure 1. long description.

This study aims to fill this gap. As summarized in Figure 1, we studied 61 PTSD patients and 62 trauma-exposed non-PTSD controls (TENC) recruited from earthquake survivors. Based on structural connectivity networks (SCNs) and functional connectivity networks (FCN) derived from multimodal MRI data, we used Laplacian eigenmode mapping to identify distinctive SFC alterations in PTSD, then explored their associations with clinical characteristics, regional molecular architecture and transcriptional profiles. We tested three hypotheses: that SFC exhibits a hierarchical organization, with PTSD patients showing regional deviations in SFC relative to TENC; that these SFC alterations correlate significantly with symptom severity as assessed by standardized PTSD scales; and that these SFC alterations correlate with relevant biological factors, including colocalization and gene expression levels.

## Methods

Figure 1a summarizes participant recruitment and testing, MR imaging and MRI data preprocessing. We describe these in turn.

### Participant recruitment and testing

Participants were enrolled in January–August 2009 from survivors of the May 2008 earthquake in Sichuan Province of China. Subjects scoring ≥35 on screening with the PTSD Checklist (PCL) (Weathers et al., 1993) were assessed with the Clinician-Administered PTSD Scale (CAPS) (Blake et al., 1995), before the Structured Clinical Interview for Diagnostic and Statistical Manual of Mental Disorders, Fourth Edition was used to confirm the PTSD diagnosis and exclude psychiatric comorbidities (First, Spitzer, Gibbon, & Williams, 1994). Survivors without PTSD but with PCL scores <35 were considered as TENC. This cohort, exposed to a common traumatic event, provides a valuable real-world context for studying trauma-related brain changes with relatively little trauma heterogeneity. Details of inclusion/exclusion criteria and evaluation are given in Supplementary Methods 1. This study was approved by the Research Ethics Committee of the West China Hospital of Sichuan University, and all subjects provided written informed consent.

The demographic and clinical data were analyzed using SPSS (v. 26.0, www.spss.com, IBM Corp., USA), with independent-sample t test for age, education, time since trauma, and PCL scores, and χ^2^ test for sex; *p* < 0.05 was taken as statistically significant.

### MRI data acquisition

Details of imaging acquisition are given in Supplementary Methods 2. In brief, high-resolution T1-weighted (T1w) images, resting-state blood oxygen level-dependent fMRI (rs-BOLD-fMRI) and DTI data were acquired using a 3 Tesla MRI scanner (EXCITE, General Electric, USA) with an eight-channel phased array head coil. Foam pads were used to reduce head motion and scanner noise, and participants were instructed to remain awake with their eyes closed during the scanning. The quality of all acquired images was evaluated by two experienced neuroradiologists.

### MRI data preprocessing

Details of preprocessing are given in Supplementary Methods 3 and 4. In brief, images were first collated into Brain Imaging Data Structure format.

Functional (rs-BOLD-fMRI) data were preprocessed using *fMRIPrep* (v. 23.1.4, https://codeocean.com/capsule/3568017/tree) (Esteban et al., 2019) with the following steps (Supplementary Methods 3): intensity nonuniformity correction, fuse and conform, skull stripping, spatial normalization, brain tissue segmentation, surface reconstruction for T1w images and generating the BOLD reference mask, head-motion estimation, susceptibility distortion correction, alignment to T1w reference, resampling for BOLD data. Then *XCP-D* (v. 0.6.0rc6, https://github.com/PennLINC/xcp_d.git) (Mehta et al., 2023), was used for high-motion exclusion, regression of nuisance covariates, despiking, band-pass filtering (0.01–0.08 Hz), and smoothing with a Gaussian kernel (FWHM 6.0 mm).

DTI data were processed using *QSIPrep* (v. 0.19.1, https://github.com/PennLINC/qsiprep.git) (Cieslak et al., 2021) in the following steps (Supplemental Methods 4): conform image and gradient orientation, group by distortion, denoising, distortion correction, head-motion correction, build subject B0 template, registration and normalization, and fiber reconstruction. These containerized pipelines automatically configure appropriate preprocessing workflows based on the data used (Gorgolewski et al., 2016), providing a unified and robust platform for best practice.

Figure 1b summarizes the construction of the functional and structural networks, and analysis of structural–functional coupling. We describe these in turn.

### FCN construction

The FCN for each subject was constructed from the preprocessed rs-BOLD-fMRI data. The whole brain was divided into 152 regions of interest (ROI), according to the Schaefer 100 atlas (Schaefer et al., 2018) supplemented with subcortical structures and cerebellum (King et al., 2019; Najdenovska et al., 2018; Pauli, Nili, & Tyszka, 2018) (Supplemental Table S1), each forming a node of the matrix. These ROIs were assigned to functional modules: these were Yeo’s seven networks (Yeo et al., 2011) (DMN, visual network [VN], frontoparietal control network [FPN], limbic network, dorsal attention network [DAN], ventral attention network [VAN], and sensorimotor network [SMN]), together with the subcortical area (SUB) and cerebellum region (CE). The averaged BOLD signals of all voxels within an ROI were defined as the value of that node; the Pearson correlation between all pairs of nodes were calculated (with Fisher’s Z-transformation to improve normality); and the normalized correlation coefficients were defined as the weights of FC (as their interpretation is controversial, negative FC were set to zero (Schwarz & McGonigle, 2011; Smith et al., 2025b). This yielded a 152 × 152 whole-brain FC matrix for each subject.

### SCN construction

The SCN for each subject was constructed from the preprocessed DTI data. After parcellation with the same atlas as for FC, the intensities of SC were defined as the number of streamlines between each pair of nodes, and each streamline was weighted to reduce fiber-tracking and region volume variability biases using the *SIFT* and *invnodevol* algorithms (R. E. Smith, Tournier, Calamante, & Connelly, 2025). We used a consistency-based threshold to reduce the effect of spurious connectivities by measuring the coefficient of variation (CV) across subjects (Roberts et al., 2017), removing the top 25% of SC with the largest CV (Baum et al., 2020). Reasoning that interregional physiological efficacies would not span a large range, we resampled the retained SC to a Gaussian distribution (mean 0.5, standard deviation 0.1) (Honey et al., 2009). This yielded a 152 × 152 whole-brain SC matrix for each subject. We performed a sensitivity analysis to evaluate the influence on our results of the specific choice of consistency-based thresholding and resampling preprocessing (Supplementary Figure S1).

### SFC analysis

SFC calculation used an eigenmode-mapping approach of proven reliability and interpretability (Facca, Del Felice, & Bertoldo, 2024; Yang et al., 2023). We performed Laplacian eigendecomposition on the normalized Laplacian matrix of each constructed SCN to obtain eigenvalue-eigenvector pairs, which were sorted in ascending order of eigenvalue. Following previous studies (Facca, Del Felice, & Bertoldo, 2024; Medaglia et al., 2018), we used a weighted sum of the top 10 low-frequency eigenmodes for each node to predict the nodal FC profile in a multilinear regression. SFC for each node is then defined as the coefficient of determination (R^2^) between the fitted FC and the empirical FC. We performed a sensitivity analysis to evaluate the influence on our results of the specific eigenmode choice (Supplementary Figure S2).

To compare SFC between PTSD and TENC, we calculated the mean between-group SFC difference for each node to generate a mean-difference map. To test whether these group differences could occur by chance, we randomly reassigned all the values into two groups and rebuilt the mean-difference maps between these, repeated 10,000 times, with α = 0.05 for a two-tailed test. We used age, gender, and years of education as covariates to reduce confounding, and FDR correction (*p* < 0.05) for multiple comparisons. We also conducted correlation analyses between SFC across all ROIs in the PTSD group and the corresponding CAPS scores using Pearson partial correlation analysis, with age, gender, and years of education included as covariates. Statistical significance was set at *p* < 0.05 after FDR correction.

Figure 1c summarizes spatial correlation analysis and partial least squares (PLS) analysis of genetic data. We describe these in turn.

### Colocalization of SFC patterns and brain tissue biological properties

To explore how the spatial patterns of SFC might be explained by the spatial distributions of underlying neurobiology, we performed spatial correlations between SFC and maps of a number of aspects of molecular, genetic, biochemical, and physiological architecture using JuSpace (v. 2.0, https://github.com/juryxy/JuSpace.git) (Dukart et al., 2021), a toolbox allowing cross-modal spatial correlation of MRI-based measures with the distribution of biologically interpretable tissue properties. Details of the maps used are given in Supplemental Table S2. They include: neurotransmitter systems (dopamine, noradrenaline, serotonin, acetylcholine, glutamate, γ-aminobutyric acid (GABA), cannabinoid, and opioid) derived from positron emission tomography or single-photon emission computed tomography; cell-type maps for glia and neurons identified from single-nucleus RNA sequencing studies, extracting Allen Human Brain Atlas (AHBA) mRNA expression values for each cell-type marker gene (Lotter et al., 2024); cerebral blood flow profiles captured by arterial spin-labeling MRI (Holiga et al., 2018); and brain mitochondrial respiratory capacity (Mosharov et al., 2025).

For each of these spatial correlation exercises, we calculated Spearman correlation coefficient between intergroup SFC alterations and the relevant map, computing exact permutation-based *p* values (with 10,000 permutations) with partial-volume-effect correction using T1 probabilities to test for significant difference from the null distribution. All analyses were corrected using FDR at *p* < 0.05. Preliminary correlation analyses revealed only a limited number of associations, consistent with the idea that the spatial distribution of molecular architecture represents stable organizational constraints rather than disease-specific drivers. We therefore further examined spatial correlations between the PTSD/TENC group-mean SFC and molecular maps using the same method.

### Relation of cortical gene expression to SFC alterations

To investigate the transcriptional basis of regional SFC changes, we assessed their association with the gene expression map using PLS regression analysis (Abdi & Williams, 2013). Details are given in Supplemental Methods 5. In brief, brain-wide microarray expression data obtained from AHBA (https://human.brain-map.org) (Hawrylycz et al., 2012) by *abagen* (v. 0.1.4 + 15.gdc4a007; https://github.com/rmarkello/abagen) (Markello et al., 2021) were used to predict the between-group SFC differences. After verification, the second component of the PLS regression (PLS2), a linear combination of gene expression values most significantly correlated with regional SFC alterations, was used for further analysis. The spin test with 10,000 random rotations was performed to assess the significance of PLS2 to control for spatial autocorrelation (Burt et al., 2020), then the PLS2 weight of each gene was evaluated for variability with 10,000 bootstrap replications, and a z-score used to rank each gene’s contribution to PLS2 was computed as the ratio of its PLS2 weight to bootstrap standard error. Only genes with FDR *p* < 0.05 were considered significant. This yielded two lists of genes: those with a significant positive correlation with regional SFC changes (PLS2+), and those with a significant negative correlation (PLS2-).

### Enrichment analysis of SFC-associated genes

Enrichment analyses were performed for significantly SFC-correlated PLS2 genes using *clusterProfiler* (v. 4.16.0; https://git.bioconductor.org/packages/clusterProfiler) (Yu, Wang, Han, & He, 2012). Gene Ontology (GO) analysis helped determine biological functions, divided into molecular functions (MFs), biological processes (BP), and cellular components (CCs) (Thomas et al., 2022). The Kyoto Encyclopedia of Genes and Genomes (KEGG) was used to discern relevant biological pathways (Kanehisa et al., 2017). Given that PLS2+ and PLS2- genes have distinct biological implications, we performed separate GO and KEGG enrichment analyses for each gene set. We used a hypergeometric test to identify ontology terms that contained more genes overlapping with PLS2+ (or PLS2-) genes than expected by chance. The human genome provided by *org.Hs.eg.db* (v. 3.21.0; https://bioconductor.org/packages/org.Hs.eg.db/) served as the background gene set. Enrichment results were considered statistically significant at *p* < 0.05 with FDR correction.

## Results

### Demographic and clinical characteristics

These are detailed in Table 1. There were no significant differences in the sex (*p* = 0.596), age (*p* = 0.894), and education (*p* = 0.629) in patients with PTSD versus TENC. As expected from the group definitions, PTSD versus TENC had significantly higher PCL (*p* < 0.001). They also had significantly lower time since trauma (*p* < 0.001).

**Table 1.** Demographic and clinical characteristics Table 1. long description.

Characteristic	PTSD (*n* = 61)	TENC (*n* = 62)	Statistics	*p*
Age (y)^a^	43.4 (10.9)	43.2 (9.6)	0.13	0.894
Sex (Female/Male)^b^	44/17	42/20	0.28	0.596
Education (y)^a^	6.8 (3.4)	7.1 (3.3)	−0.49	0.629
Time since trauma (m)^a^	10.5 (2.0)	13.5 (0.9)	−10.84	< 0.001
PCL score^a^	51.5 (11.0)	25.8 (4.7)	16.80	< 0.001
CAPS score	60.3 (13.3)	–	–	–

*Note:* Data presented as *n* or mean (standard deviation). Abbreviations: CAPS, Clinician-Administered PTSD Scale; PCL, PTSD Checklist; PTSD, post-traumatic stress disorder; TENC, trauma-exposed non-PTSD controls. ^a^ By two sample *t* test; ^b^ by Chi-square test.

### Network-specific divergence of SFC in PTSD

**Figure 2.**
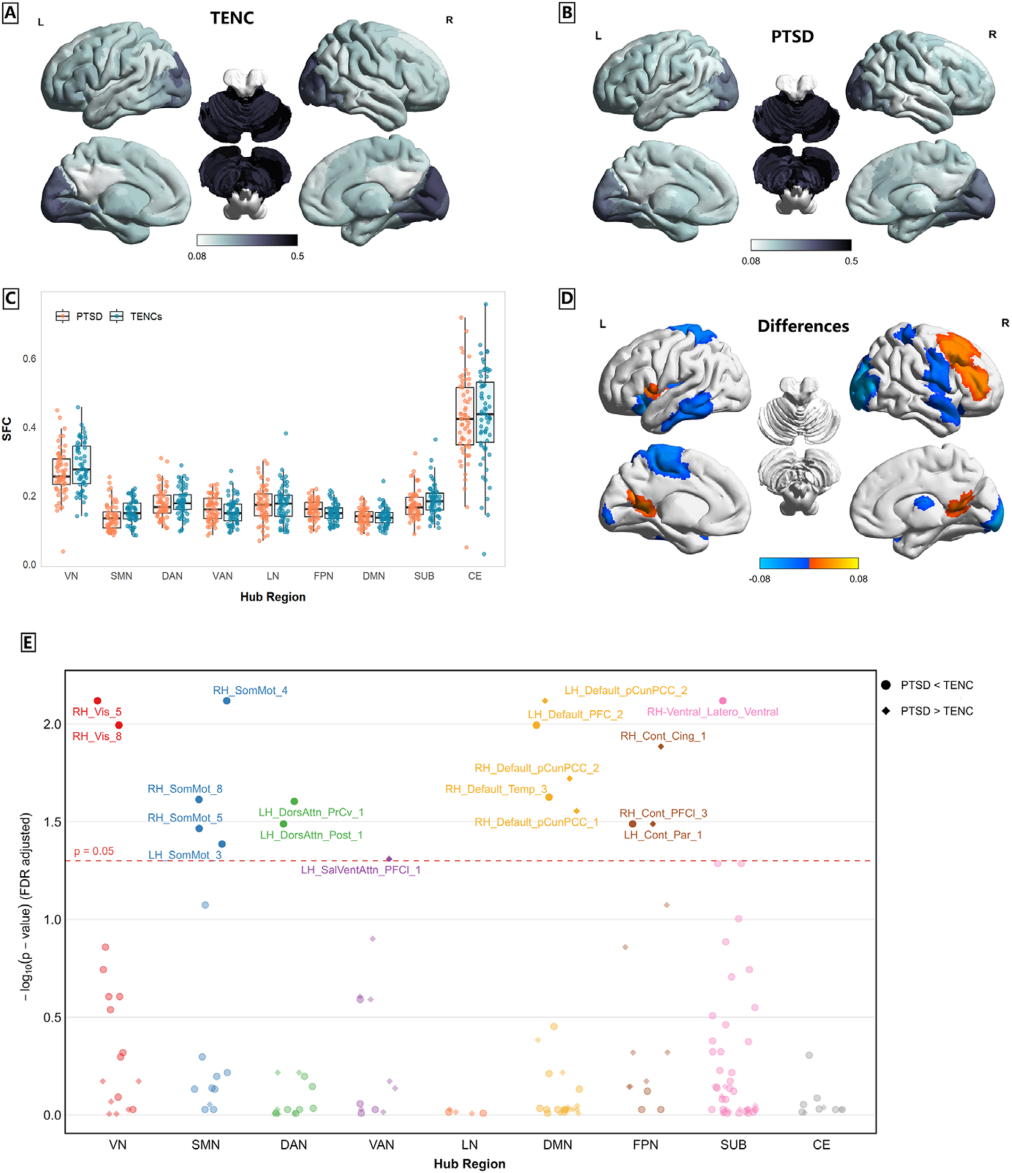
Structure–function coupling distribution and group difference. (a) and (b) Map structure–function coupling (SFC) in trauma-exposed non-PTSD controls (TENC) and PTSD patients, respectively (see gray-scale key). (c) A boxplot of network-level SFC values in the PTSD (orange) and TENC (blue) groups; each data point represents a subject. (d) Maps the significant (*p* < 0.05, false discovery rate corrected) node-level group differences of SFC, both positive and negative (see color key). (e) These node-level differences grouped by network; symbol shape denotes the direction of group difference (see key); each data point represents a node, and nodes showing significant group differences are labeled. Abbreviations: CE, cerebellum region; DAN, dorsal attention network; DMN, default mode network; FPN, frontoparietal control network; LN, limbic network; SMN, sensorimotor network; SUB, subcortical area; VAN, ventral attention network; VN, visual network. Figure 2. long description.

Across the whole brain, the SFC distribution varied considerably (overall range TENC: 0.087–0.485, PTSD: 0.106–0.479) (Figure 2a,b). At the network level, both groups showed notably high SFC in the cerebellum (mean [SD]: TENC: 0.433 [0.134], PTSD: 0.423 [0.121]) and VN (TENC: 0.285 [0.069], PTSD: 0.265 [0.069]), but lower values in the other networks; there were no significant between-group differences (Figure 2c and Supplementary Table S3). At the node level, patients with PTSD showed significantly altered SFC in 18 ROIs compared with TENC (all $p_{fdr}$ < 0.05, Figure 2d,e, Table 2). At the node level, patients with PTSD showed significantly altered SFC in 18 ROIs compared with TENC (all $p_{fdr}$ < 0.05, Figure 2d,e, Table 2): lower SFC compared with TENC at regions in the DAN, VN, SMN, and thalamus presumably reflects abnormalities in the attentional control and sensory integration systems; conversely, SFC was higher compared with TENC in the VAN; and the DMN and FPN, both transmodal networks, showed differentiated bidirectional alterations. This regional heterogeneity suggests that PTSD involves complex within-network functional reorganization.

**Table 2.** Significant node-level between-group differences in structure–function coupling Table 2. long description.

Region	Network	PTSD	TENC	Mean difference	*p*
LH_SomMot_3	SMN	0.136 (0.056)	0.166 (0.062)	−0.031	0.041
RH_SomMot_4	SMN	0.115 (0.048)	0.149 (0.044)	−0.034	0.008
RH_SomMot_5	SMN	0.127 (0.061)	0.158 (0.056)	−0.031	0.034
RH_SomMot_8	SMN	0.149 (0.054)	0.183 (0.061)	−0.034	0.024
RH_Vis_5	VN	0.293 (0.093)	0.362 (0.109)	−0.069	0.008
RH_Vis_8	VN	0.289 (0.095)	0.362 (0.112)	−0.073	0.010
LH_DorsAttn_Post_1	DAN	0.167 (0.072)	0.211 (0.085)	−0.044	0.032
LH_DorsAttn_PrCv_1	DAN	0.178 (0.053)	0.212 (0.057)	−0.034	0.025
LH_SalVentAttn_PFCl_1	VAN	0.182 (0.059)	0.151 (0.062)	0.032	0.049
LH_Cont_Par_1	FPN	0.145 (0.048)	0.176 (0.061)	−0.031	0.032
RH_Cont_PFCl_3	FPN	0.186 (0.059)	0.154 (0.057)	0.032	0.032
RH_Cont_Cing_1	FPN	0.135 (0.048)	0.105 (0.043)	0.031	0.013
LH_Default_PFC_2	DMN	0.168 (0.057)	0.223 (0.074)	−0.056	0.010
LH_Default_pCunPCC_2	DMN	0.126 (0.039)	0.088 (0.034)	0.038	0.008
RH_Default_Temp_3	DMN	0.106 (0.046)	0.135 (0.051)	−0.029	0.024
RH_Default_pCunPCC_1	DMN	0.130 (0.049)	0.099 (0.056)	0.030	0.028
RH_Default_pCunPCC_2	DMN	0.114 (0.043)	0.087 (0.035)	0.027	0.019
RH-Ventral_Latero_Ventral	SUB	0.129 (0.057)	0.148 (0.062)	−0.057	0.008

*Note:* False discovery rate correction (*p* < 0.05) applied for correction of multiple comparisons. Abbreviations: DAN, dorsal attention network; DMN, default mode network; FPN, frontoparietal control network; PTSD, post-traumatic stress disorder; SMN, sensorimotor network; SUB, subcortical area; TENC, trauma-exposed non-PTSD controls; VAN, ventral attention network.

**Figure 3.**
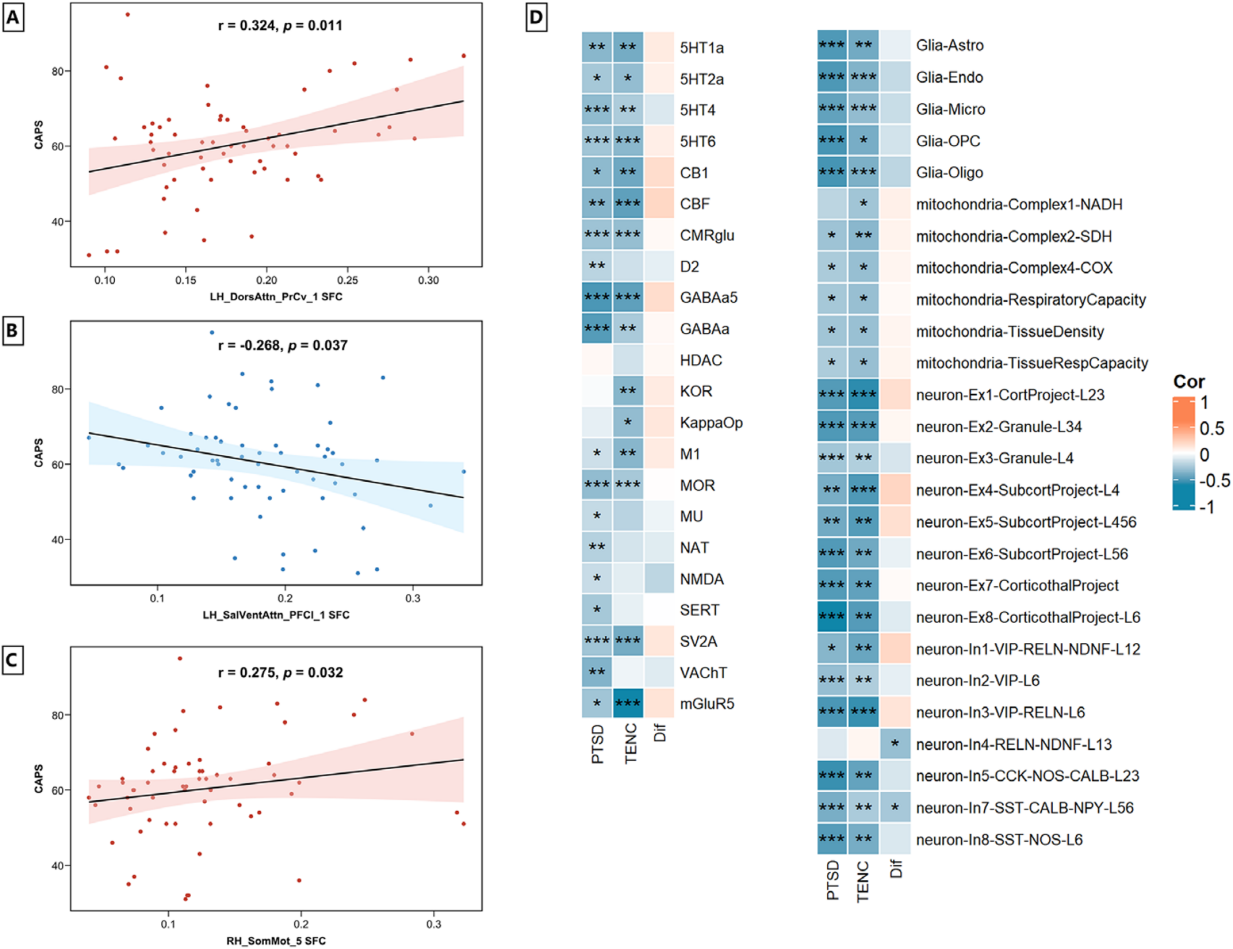
Partial correlation and spatial colocalization analysis. (a–c) Scatterplots showing associations (not significant after FDR correction) in PTSD patients between the Clinician-Administered PTSD Scale score (CAPS) and structure–function coupling (SFC) at three nodes: positive correlation for (a) LH_DorsAttn_PrCv_1 (part of DAN) and (c) RH_SomMot_5 (part of SMN), and negative correlation for (b) LH_SalVentAttn_PFCl_1 (part of VAN). Each data point represents a patient. (d) The spatial correlations (see color key) of the SFC distribution of PTSD patients (first columns), trauma-exposed non-PTSD controls (TENC) (second columns) and the PTSD-TENC group difference (third columns) with various neurotransmitter, cell-type, and mitochondria maps, as labeled (* *p* < 0.05, ** *p* < 0.01, *** *p* < 0.001, false discovery rate corrected). Figure 3. long description.

Using partial correlation, in the PTSD group CAPS scores were correlated with nodal SFC of 3 of the 18 regions which showed significant between-group differences in Table 2: positive correlation for RH_SomMot_5 (*r* = 0.275, *p* = 0.032) and LH_DorsAttn_PrCv_1 (*r* = 0.324, *p* = 0.011), and negative correlation for LH_SalVentAttn_PFCl_1 (*r* = −0.268, *p* = 0.037) (Figure 3a–c). There were also associations between regional SFC and CAPS scores in other ROIs that did not show significant between-group differences, including regions in the DMN and SUB (Supplementary Figure S3). However, none of these findings survived FDR correction, and must therefore be regarded as exploratory.

### SFC correlations with brain tissue biological properties

The SFC maps were negatively correlated with the spatial distribution maps of various molecular structures (all $p_{fdr}$ < 0.05 for both groups), including glutamate, serotonin, acetylcholine, noradrenaline, cannabinoid, GABA, metabolism, excitatory/inhibitory neurons, glia, and mitochondria maps in both PTSD and TENC (Figure 3d, first and second columns, respectively). In the between-group SFC difference map (Figure 3d, third columns), the only significant correlations were two inhibitory neuron maps (In4: rho = −0.31, $p_{fdr}$ = 0.014; In7: rho = −0.27, $p_{fdr}$ = 0.035).

### SFC associations with gene expression

**Figure 4.**
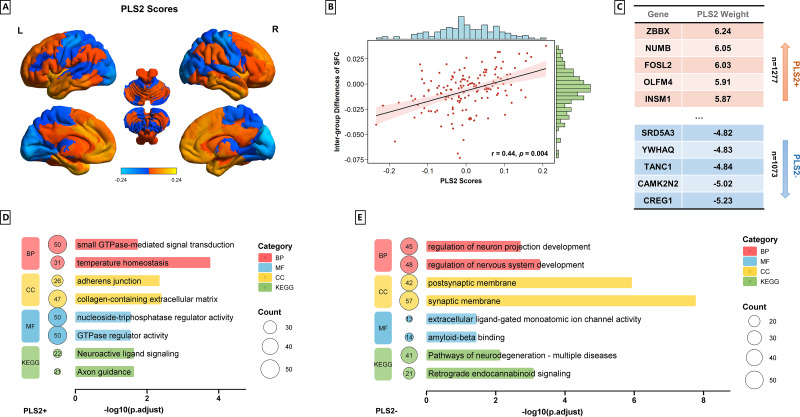
Transcriptional correlation analysis. (a) Maps the weighted gene expression (see color key) of the second component of the partial least squares regression (PLS2) scores. (b) A scatterplot (with histograms on the x- and y-axes) showing the positive correlation between average node-level intergroup differences of structure–function coupling (SFC) and PLS2 scores (*p* = 0.004, spin test corrected). (c) The ranked PLS2 gene weights (*p* < 0.05, false discovery rate corrected), both positive and negative (see color key). The top five genes of each direction are listed. (d) and (E) The enrichment of PLS2- and PLS2+ genes significantly (*p* < 0.05, false discovery rate corrected) associated with SFC alterations in PTSD versus TENC; gene functions are as labeled, classified (see color key) into molecular functions (MFs), biological processes (BPs), cellular components (CC), and Kyoto Encyclopedia of Genes and Genomes (KEGG); count given in the coded circles. Figure 4. long description.

In the PLS regression analysis, PLS2 explained 19% of the variance ( $p_{spin}$ = 0.006) in SFC, and the map of the PLS2 weight reflects a broadly anterior-to-posterior gradient of gene expression (Figure 4a). PLS2-weighted gene expression was positively correlated with intergroup SFC differences (*r* = 0.44, $p_{spin}$ = 0.004, Figure 4b). Ranking the normalized PLS2 weights using univariate z-tests, and dividing into genes showing positive (PLS2+) and negative (PLS2-) correlations, we identified 1277 PLS2+ (z > 2.68) and 1073 PLS2- (*z* < −2.68) genes significantly related to intergroup SFC changes (all $p_{fdr}$ < 0.05, Figure 4c). PLS2+ genes are overexpressed in regions where SFC is higher in PTSD versus TENC, while PLS2- genes are underexpressed in those regions.

### Gene enrichment associations with SFC

PLS2- genes associated with SFC differences were most enriched for BP in regulation of neuron projection and nervous system development, for CC in synaptic and postsynaptic membrane, and for MF in extracellular ligand-gated monoatomic ion channel activity and amyloid-beta binding. Conversely, PLS2+ genes were primarily enriched for BP related to small GTPase-mediated signal transduction and temperature homeostasis, for CC involving adherens junctions and collagen-containing extracellular matrix, and for MF associated with nucleoside-triphosphatase regulator activity and GTPase regulator activity. KEGG analysis revealed significant enrichment in pathways of neurodegeneration-multiple diseases and retrograde endocannabinoid signaling pathway for PLS2- genes, and in neuroactive ligand signaling and axon guidance pathway for PLS2+ genes (Figure 4d,e and Supplemental Table S4).

## Discussion

Using a multimodal imaging fusion method based on Laplace eigendecomposition, we characterized for the first time abnormal patterns of whole-brain-scale SFC in PTSD patients, and revealed their relationship to multidimensional brain biology. The participant cohort used in this study has been previously described in several publications focusing on identifying imaging biomarkers of PTSD subjects, although in a single-modal fashion (Lei et al., 2015; Li et al., 2016; Li et al., 2016, 2021). While these studies provided insights into specific aspects of PTSD neuropathology, they were inherently limited by their single-modal experimental design. Going beyond this, the present work investigates potential PTSD biomarkers from a new perspective by combining structural and functional images to identify changes in SFC, and then linking these to the underlying molecular architecture. This new analysis yields a deeper understanding of the pathogenesis and molecular mechanisms of PTSD. We have shown that PTSD patients have region-specific alterations in SFC across multiple brain regions, some of which are significantly correlated with the severity of PTSD; the regional distribution pattern of SFC is associated with the spatial distribution of biological properties of brain tissue; and genes associated with altered SFC in PTSD are enriched in various BPs related to neural development and neural signaling. These findings provide insights into the structural and functional interactions and network wiring rules of PTSD and offer potential biomarkers for diagnostic and therapeutic research.

The integrated measure SFC contains more information than the sum of its structural and functional components, and so variations in SFC may arise from diverse neurobiological mechanisms, the interpretation of which depends on the disease context and clinical circumstances. Rather than being directly mapped onto a single mechanism, increases or decreases in SFC are better understood as a manifestation of rebalancing between structural constraints and functional demands within brain networks (Fotiadis et al., 2024). To define their complex interdependencies we used the eigendecomposition approach, which models functional signals as a diffusion process on the underlying structural network (Abdelnour, Voss, & Raj, 2014). In predicting FC through eigenmode mapping of SC, we simulate how neural activity propagates to form the observed functional patterns. This physics framework, grounded in topology, enhances the neurobiological interpretability of SFC (Abdelnour et al., 2018). As in earlier studies using this approach (Facca, Del Felice, & Bertoldo, 2024; Wang, Owen, Mukherjee, & Raj, 2017), a small number of eigenmodes sufficed for accurate FC reconstruction, which improves sensitivity to subtle changes, and simplifies investigation of structure–function relationships in disease (Lu et al., 2025).

We found heterogeneous whole-brain SFC distribution aligning with established hierarchies (Baum et al., 2020; Vázquez-Rodríguez et al., 2019): the gradient of gradual SFC decoupling from unimodal (VN, SMN, and SUBs) to transmodal regions (DMN, FPN, and VAN) reflects decreasing functional specialization from modality-specific processing to cognitive flexibility (Preti & Van De Ville, 2019). Areas with relatively high SFC, including the cerebellum, have high heritability (Gu, Jamison, Sabuncu, & Kuceyeski, 2021). Consequently, high SFC not only reflects structure-constrained variation in dynamics (Fernandez-Iriondo et al., 2021), but also underpins stability across individuals and generations.

Group comparisons revealed multiple regional SFC alterations. Among the higher-order networks, both DMN and FPN showed extensive SFC changes, consistent with their known roles in PTSD (Alexandra Kredlow et al., 2022; Bao et al., 2021; Patriat, Birn, Keding, & Herringa, 2016). DMN abnormalities overlapped with earlier findings from graph theory in the same cohort (Lei et al., 2015), while SFC alterations in right FPN may be related to excessive threat attention bias in PTSD (Berg et al., 2021), a further manifestation of dysregulated fear and threat control. PTSD also showed lower SFC in the right visual regions and thalamus. The thalamus is thought to modulate activity across fear-processing regions during high-threat states (Venkataraman & Dias, 2023), and amygdala–thalamus connectivity is correlated with PTSD severity (Ben-Zion et al., 2020). Threatening visual signals are transmitted to the amygdala via the superior colliculus and occipital lobe (Koller, Rafal, Platt, & Mitchell, 2019), and this circuit takes part in fear learning (Wei et al., 2015). Notably, variability in threat-visual circuitry connectivity partially underlies susceptibility to PTSD (Harnett et al., 2025), and intervention in high visuospatial demand tasks following trauma can alleviate intrusive painful memories (Iyadurai et al., 2018), so treatment targeting visual neural circuits shows promise. In addition, PTSD patients with lower VAN connectivity show clinically significant attentional impairment (Esterman et al., 2020). In connectome gradient analysis, PTSD patients show higher system segregation in SMN and lower participation coefficients in DAN, correlating with global gradient variance and CAPS scores (He et al., 2025). The observed alterations in SFC within these network regions suggest corresponding functional abnormalities.

It is of course difficult to establish causation in cross-sectional studies. Due to statistical power limitations, the associations between regional SFC and clinical measures across the SMN, VAN, DAN, DMN, and SUB did not survive correction for multiple comparisons. Nevertheless, these findings also provide valuable insights into the potential contribution of SFC alterations to the neural mechanisms underlying PTSD. On the one hand, network configuration abnormalities reflected by SFC may underlie core PTSD symptomatology (Lanius et al., 2015; Nicholson et al., 2020). For instance, functional alterations in the DMN have been associated with excessive self-referential processing, which may facilitate the intrusive recollection of traumatic memories, manifesting as flashbacks and reexperiencing symptoms (Lanius, Terpou, & McKinnon, 2020). Similarly, dysregulated modulation of attention-related networks may lead to attentional rigidity, potentially accounting for threat hypersensitivity and hypervigilance observed in PTSD patients (Koch et al., 2016). On the other hand, these findings also suggest that PTSD exhibits clinical heterogeneity that can obscure group-level effects and the directionality of associations (Ben-Zion et al., 2023; Jiang et al., 2024), highlighting the need for future studies with larger samples to identify potential biological subtypes of PTSD. In any case, more information on these regions may improve our understanding of PTSD severity trajectories.

Spatial colocalization analysis demonstrated broad negative correlations between SFC and various aspects of brain tissue biology: specifically, regions enriched for neurons, glia, neurotransmitter systems, and metabolic activity show lower SFC. This may reflect the greater functional flexibility enabled by complex microcircuitry and higher metabolic demands (Chen et al., 2023; Luppi et al., 2022). PTSD pathophysiology involves various substrates: recent high-throughput single-cell sequencing studies implicate dysregulation in inhibitory neurons, endothelial cells, and microglia across multiple neurotransmitter pathways (Hwang et al., 2025). Interestingly, in our study, spatial SFC–neurobiological correlations were highly consistent between PTSD and TENC, with only weak correlations with intergroup SFC differences. Cortical chemoarchitecture shares key organizational traits with functional and structural brain anatomy (Hänisch et al., 2023), and a recent study attributed cortical SFC variability to the overlapping distributions of various neurotransmitter systems (L. Jiang et al., 2025). Our results may therefore shed new light on the biological substrate of SFC spatial heterogeneity: intrinsic cellular and molecular architecture provides a fundamental constraint framework for the distribution of SFC, persistent regardless of disease status. They also support the idea that the variability in the relationship between white matter structure and neural activity constitutes an inherent property of brain networks (Fotiadis et al., 2024).

Finally, gene association analysis revealed significant correlations between PTSD-related SFC alterations and PLS2 gene co-expression patterns. Brain genes showed differential expression across regions characterized by varying SFC profiles. PLS2- genes were predominantly enriched in terms related to neuronal synapses, neurodegenerative disease pathways, and ion channel activity. Proper development and regulation of neuron are essential for constructing accurate neural circuits (Tau & Peterson, 2010). The widespread lower SFC observed in PTSD may be associated with dysregulation of synaptic regulatory genes, potentially contributing to anatomical–functional decoupling, while alterations in ion channel activity further disrupt synaptic information transmission. These findings resonate with recent large-scale transcriptomic analyses of postmortem brain tissue from PTSD patients, which identified downregulation in a co-regulated set of genes marking interneuron function (Girgenti et al., 2021), raising the possibility that SFC-associated gene patterns may reflect pathological synaptic dysfunction in PTSD. Abnormalities in the endocannabinoid signaling pathway, which have been reported in PTSD patients, may promote the retention of aversive emotional memories (Hill et al., 2013). Furthermore, enrichment in amyloid-beta-binding functions suggests the involvement of molecules participating in neurotoxicity and protein aggregation, offering a potential link to chronic neurodegenerative mechanisms underlying PTSD (Kritikos et al., 2023). By contrast, PLS2+ genes showed enrichment patterns suggestive of compensatory signaling mechanisms in PTSD. As key regulators of cytoskeletal dynamics, GTPase-related proteins directly participate in synaptic structural and functional plasticity (Hedrick et al., 2016). Neuroplasticity enables the brain to reorganize and adapt, enhancing resilience to trauma and adversity (Udeh-Momoh et al., 2025). It is known that genetic variants can increase trauma vulnerability, and that a supportive environment can mitigate this effect (Clukay et al., 2019). Collectively, these findings suggest that transcriptional profiles, being more plastic and responsive to environmental and pathological processes, better capture abnormalities in brain organization, and may be responsible for SFC alterations. However, it is important to note that the enriched pathways identified here are relatively broad and that the interpretations remain speculative at this stage. Therefore, our findings provide exploratory transcriptomic correlates of SFC alterations that suggest candidate molecular pathways that may interact with trauma-related pathophysiology and warrant further mechanistic investigation.

This study has some limitations. First, the cross-sectional design precluded determination of whether the SFC alterations represent trait-like or state-like effects, and their dynamic evolution throughout PTSD progression. Second, given the heterogeneity in trauma exposures and individual baselines, the generalizability of findings from our relatively limited sample size requires verification, ideally combining longitudinal follow-up with individual deep phenotyping. Third, in our sensitivity analyses (Supplementary Figures S1, S2) the choice of parameters in constructing SFC has a non-negligible influence on the analytical outcomes, no doubt largely due to necessary trade-offs in the methodological pipelines, familiar from other studies (Botvinik-Nezer et al., 2020; Schilling et al., 2021; Zhou et al., 2022). The sensitivity analyses also make an indirect argument for the brain network perspective (Osmanlıoğlu, Alappatt, Parker, & Verma, 2020): unimodal cortical regions are generally quite sensitive to parameter selection, while ROIs distributed in transmodal association cortices are more robust. This makes sense in terms of the hierarchical organization of brain networks: neural activity in primary sensory areas is more tightly coupled to local anatomical structures, while higher-order association cortices support functional integration through distributed networks, thus depending more on global properties. In the absence of a definitive methodological consensus, we adopted parameter ranges used in similar studies (Baum et al., 2020; Facca, Del Felice, & Bertoldo, 2024; Honey et al., 2009). Future research should systematically evaluate the potential effects of methodological variability on SFC estimation. Fourth, the spatial neurobiological maps and AHBA gene expression data originated from distinct healthy cohorts, which constrains interpretation of their correlations with SFC. Future analyses would benefit from multimodal data acquisition within the same participant population. Fifth, while spatial colocalization and gene association analyses provide valuable insights into neurobiology and brain organization in neuroimaging contexts, inferences remain at a correlative, noncausal level. Precise regulatory mechanisms necessitate further validation using animal models and molecular studies.

## Conclusion

This study explored SFC deviations associated with PTSD. We found that PTSD, compared to TENC, is characterized by extensive alterations in SFC across multiple brain networks, including SMN, VN, DMN, DAN, VAN, FPN, and thalamus, and that the aberrant SFC patterns are associated with specific molecular and transcriptomic profiles. By integrating macroscale *in vivo* imaging with microscale biological substrates, our results advance the understanding of SFC in the psychopathology of PTSD and may help to establish targeted diagnosis and treatment measures.

## Supporting information

10.1017/S0033291726104838.sm001 Song et al. supplementary material Song et al. supplementary material

## Data Availability

The data supporting the findings of this study are available on reasonable request from the corresponding author.

## Supplementary material

The supplementary material for this article can be found at http://doi.org/10.1017/S0033291726104838.

## Long descriptions

### Figure 1. Long description

The flowchart is divided into three sections labeled A, B, and C.

Panel A, Participant and scanning workflow, starts at the top with a Checklist plus Diagnosis icon. An arrow points down to two groups of people icons: P T S D, N equals 61, and T E N C, N equals 62. A downward arrow leads to a T 1 plus B O L D plus D T I Scanning icon. The final step at the bottom shows three brain scan cross-sections labeled Preprocessing.

Panel B, Connectivity and structure-function coupling analysis, begins with two brain imaging types on the left: D T I and f M R I. Arrows from both point to a central Atlas with 152 regions. From the Atlas, arrows point right to two sets of heatmaps: S C matrices and F C matrices, each labeled sub 1, sub 2, through sub n. A large arrow labeled Eigenmode mapping points from these matrices to a vertical bar chart representing S F C for each subject.

Panel C, S F C annotation and analysis, features a central brain model labeled Group difference. Above it, three biological maps labeled Cell type, Neurotransmitter, and Mitochondria are linked to S F C via a double-headed arrow labeled Spatial correlation. Below the brain model, a large matrix labeled A H B A with 152 regions and 15677 genes is linked via an arrow labeled P L S. This leads to a final step labeled Enrichment analysis, which points to a dot plot representing G O and K E G G pathways.

Navigate back to Figure 1..

### Table 1. Long description

The table consists of five columns: Characteristic, P T S D (n equals 61), T E N C (n equals 62), Statistics, and p-value.

* Age in years: P T S D 43.4 (10.9), T E N C 43.2 (9.6), Statistics 0.13, p equals 0.894.

* Sex (Female/Male): P T S D 44/17, T E N C 42/20, Statistics 0.28, p equals 0.596.

* Education in years: P T S D 6.8 (3.4), T E N C 7.1 (3.3), Statistics negative 0.49, p equals 0.629.

* Time since trauma in months: P T S D 10.5 (2.0), T E N C 13.5 (0.9), Statistics negative 10.84, p is less than 0.001.

* P C L score: P T S D 51.5 (11.0), T E N C 25.8 (4.7), Statistics 16.80, p is less than 0.001.

* C A P S score: P T S D 60.3 (13.3), with no data provided for the T E N C group.

Note: Data is presented as n or mean with standard deviation. P C L stands for P T S D Checklist and C A P S stands for Clinician-Administered P T S D Scale.

Navigate back to Table 1..

### Figure 2. Long description

Panel A and B show brain maps for T E N C and P T S D groups. Both display S F C values ranging from 0.08 light blue to 0.5 dark purple. High coupling is concentrated in the posterior and cerebellar regions. Panel C is a boxplot with Hub Region on the x-axis and S F C on the y-axis from 0.0 to 0.6. It compares P T S D orange and T E N C blue across nine networks: V N, S M N, D A N, V A N, L N, F P N, D M N, S U B, and C E. The C E network shows the highest S F C values for both groups. Panel D maps node-level differences from negative 0.08 blue to positive 0.08 orange. Significant differences are visible in the frontal and parietal lobes. Panel E is a scatter plot of Hub Regions versus negative log 10 p-value adjusted. A red dashed line at p equals 0.05 marks the significance threshold. Points above this line are labeled by region and direction. Circles indicate P T S D less than T E N C, and diamonds indicate P T S D greater than T E N C. Significant nodes include R H underscore Vis underscore 5, R H underscore SomMot underscore 4, L H underscore Default underscore pCunPCC underscore 2, and R H underscore Cont underscore Cing underscore 1.

Navigate back to Figure 2..

### Table 2. Long description

The table consists of 6 columns and 18 data rows. The columns are: Region, Network, P T S D (mean and standard deviation), T E N C (mean and standard deviation), Mean difference, and p-value.

Key data points include:

* S M N Network: Four regions (L H SomMot 3, R H SomMot 4, 5, and 8) show negative mean differences ranging from -0.031 to -0.034, with p-values between 0.008 and 0.041.

* V N Network: R H Vis 5 and R H Vis 8 show the largest negative mean differences of -0.069 and -0.073 respectively, both with p-values ≤ 0.010.

* D A N Network: L H DorsAttn Post 1 and PrCv 1 show negative mean differences of -0.044 and -0.034.

* V A N Network: L H SalVentAttn P F C l 1 shows a positive mean difference of 0.032 (p = 0.049).

* F P N Network: Three regions show mixed results; L H Cont Par 1 is negative (-0.031), while R H Cont P F C l 3 and Cing 1 are positive (0.032 and 0.031).

* D M N Network: Five regions are listed. L H Default P F C 2 and R H Default Temp 3 have negative differences (-0.056 and -0.029). L H Default pCunP C C 2, R H Default pCunP C C 1, and R H Default pCunP C C 2 have positive differences ranging from 0.027 to 0.038.

* S U B Network: R H Ventral Latero Ventral shows a negative mean difference of -0.057 (p = 0.008).

All p-values are less than 0.05, indicating statistical significance after false discovery rate correction.

Navigate back to Table 2..

### Figure 3. Long description

Panel A is a scatterplot with red data points showing a positive linear trend between L H underscore Dors Attn underscore Pr Cv underscore 1 S F C on the x-axis and C A P S on the y-axis, with r equals 0.324 and p equals 0.011. Panel B shows a negative linear trend with blue data points for L H underscore Sal Vent Attn underscore P F C l underscore 1 S F C, with r equals negative 0.268 and p equals 0.037. Panel C shows a positive linear trend with red data points for R H underscore Som Mot underscore 5 S F C, with r equals 0.275 and p equals 0.032. Panel D is a large heatmap divided into two main vertical sections. Each section has three columns labeled P T S D, T E N C, and Dif. The color scale ranges from blue (negative 1) to orange (1). The left section lists neurotransmitter receptors and transporters such as 5 H T 1 a, 5 H T 2 a, C B 1, G A B A a, and m Glu R 5. The right section lists cell types and mitochondria markers including Glia-Astro, Glia-Micro, mitochondria-Complex 1-N A D H, and various excitatory (Ex) and inhibitory (In) neuron subtypes. Asterisks indicate significance levels: one asterisk for p less than 0.05, two for p less than 0.01, and three for p less than 0.001. Most correlations in the P T S D and T E N C columns are negative (blue), while the Dif column shows mostly neutral or slightly positive correlations.

Navigate back to Figure 3..

### Figure 4. Long description

Panel A shows four cortical views and two subcortical views of a brain mapping P L S 2 scores. The color scale ranges from negative 0.24 in blue to positive 0.24 in orange. Panel B is a scatterplot with P L S 2 scores on the x-axis and Inter-group Differences of S F C on the y-axis. A linear regression line shows a positive correlation with r equals 0.44 and p equals 0.004. Marginal histograms are located on the top and right edges. Panel C is a table of ranked P L S 2 gene weights. The top five positive genes in orange are Z B B X, N U M B, F O S L 2, O L F M 4, and I N S M 1. The top five negative genes in blue are S R D 5 A 3, Y W H A Q, T A N C 1, C A M K 2 N 2, and C R E G 1. Panels D and E are horizontal bar charts for P L S 2 plus and P L S 2 minus gene enrichment. The x-axis represents negative log 10 p-adjust. Categories are color-coded: B P in red, C C in yellow, M F in blue, and K E G G in green. Circles next to each bar contain the gene count. In Panel D, the highest enrichment is for small G T P ase-mediated signal transduction. In Panel E, the highest enrichment is for synaptic membrane and postsynaptic membrane.

Navigate back to Figure 4..
