# Supplementary material for: Variations of structural–functional coupling in post-traumatic stress disorder are associated with underlying molecular and transcriptional features
Source: Psychol Med. 2026 Jun 18;56:e196. doi: 10.1017/S0033291726104838 (PMC13280688; doi:10.1017/S0033291726104838)
Supplement: Song et al. supplementary material [file S0033291726104838sup001.docx]

**Supplemental Material**

[**Supplemental Methods** 2](#_Toc228441928)

[1. Participants 2](#_Toc228441929)

[2. Image acquisition 2](#_Toc228441930)

[3. Functional (rs-BOLD-fMRI) data preprocessing 3](#_Toc228441931)

[4. Structural (DTI) data preprocessing 5](#_Toc228441932)

[5. Gene expression data processing 6](#_Toc228441933)

[**Table S1. Definition of the 152 regions of interest (ROI)** 7](#_Toc228441934)

[**Table S2. Spatial colocalization maps** 9](#_Toc228441935)

[**Table S3. Network level between-group differences in structure-function coupling** 10](#_Toc228441936)

[**Table S4. Results of enrichment analysis** 11](#_Toc228441937)

[**Figure S1. Group-wise differences using different thresholds (Panels A-J) and normalization (Panel K), and statistically significant regions common to all tested parameters (Panel L)** 12](#_Toc228441938)

[**Figure S2. Group-wise differences using different eigenmodes (Panel A-K), and statistically significant regions common to all tested parameters (Panel L)** 13](#_Toc228441939)

[**Figure S3. Results of partial correlation analysis in non-significant brain regions** 14](#_Toc228441940)

[**References** 15](#_Toc228441941)

**Supplemental Methods**

# Participants

Participants were enrolled between January and August 2009 from survivors of the 8.0 magnitude earthquake in Sichuan Province of China in May 2008. At interview they were screened with the PTSD Checklist (PCL) (Weathers, Litz, Herman, Huska & Keane, 1993); those scoring ≥35 in PCL were assessed by the Clinician-Administered PTSD Scale (CAPS) (Blake et al., 1995), then the Structured Clinical Interview for DSM-IV (Diagnostic and Statistical Manual of Mental Disorders, Fourth Edition) was used to confirm the PTSD diagnosis and exclude psychiatric comorbidities (First, Spitzer, Gibbon, & Williams, 1994). Survivors scoring PCL <35, and those who did not meet diagnostic criteria for PTSD diagnosis, were considered as trauma-exposed non-PTSD controls (TENC).

Inclusion criteria were: personally experienced the earthquake and witnessed death, serious injury, or the collapse of buildings; suffered no physical injury, including head injury, or >5 min loss of consciousness; had no PTSD diagnosis before the earthquake; received no psychopharmacologic treatment or psychologic interventions before the MRI scan; age ≥18 years; right-handed.

A large-scale survey of 4200 survivors identified 415 eligible subjects with PTSD and 109 TENC. Subjects were excluded if they met any of these criteria: other psychiatric comorbidities, early life trauma exposure and/or alcohol abuse (n =134); current or history of brain injury (n =12); other clinically important medical or neurologic conditions (n =58); any MRI contraindication (n =81); left-handed (n =16); brain lesions identified on MRI (n =5); age <18 years (n =24); unavailable data (n =10); excessive head motion rendering MRI data unsuitable for processing (n =14).

This study was approved by the Research Ethics Committee of the West China Hospital of Sichuan University. All subjects provided written informed consent.

# Image acquisition

MRI data were acquired at the Department of Radiology in West China Hospital using a 3 Tesla MRI scanner (EXCITE, General Electric, USA) with an 8‐channel phased array head coil. Foam pads were used to reduce head motion and scanner noise.

High‐resolution T1‐weighted images were acquired with a sagittal three‐dimensional spoiled gradient recall sequence with repetition time (TR) 8.5 ms, echo time (TE) 3.4 ms, inversion time 400 ms, slice thickness 1 mm without gap, 156 axial slices, matrix size 256 × 256, field of view (FOV) 240 × 240 mm^2^, and flip angle 12°.

Diffusion tensor imaging (DTI) data were obtained with 15 noncollinear directions (b = 1000 s/mm^2^) plus a reference image without diffusion weighting (b = 0), TR 12000 ms, TE 70.8 ms, slice thickness 3 mm without gap, matrix size 128 × 128, and FOV 240 × 240 mm^2^.

For resting-state blood oxygen level-dependent functional MRI (rs-BOLD-fMRI), participants were instructed to remain awake with eyes closed during scanning, then 200 gradient-echo echo-planar imaging volumes were acquired with TR 2000 ms, TE 30 ms, slice thickness 5 mm without gap, 30 axial slices per volume, matrix size 64 × 64，FOV 240 × 240 mm^2^, flip angle 90°, and voxel size 3.75 × 3.75 × 5 mm^3^.

# Functional (rs-BOLD-fMRI) data preprocessing

rs-BOLD-fMRI data was preprocessed using the pipeline *fMRIPrep* 23.1.4 (Esteban et al., 2019), which is based on *Nipype* 1.8.6 (Gorgolewski et al., 2011). This requires the input dataset to be in Brain Imaging Data Structure (BIDS) format, and to include at least 1 (in this case exactly 1) T1-weighted (T1w) structural image and a BOLD series. The T1w image was corrected for intensity non-uniformity (INU) with *N4BiasFieldCorrection* (Tustison et al., 2010), distributed with ANTs (Avants, Epstein, Grossman, & Gee, 2008), and used as T1w-reference throughout the workflow. The T1w-reference was skull-stripped with a *Nipype* implementation of the *antsBrainExtraction.sh* workflow (from ANTs), using OASIS30ANTs as target template. Segmentation of cerebrospinal fluid (CSF), white-matter (WM) and gray-matter (GM) was performed on the brain-extracted T1w using *fast* in the FMRIB Software Library (FSL) (Zhang, Brady, & Smith, 2001). Brain surfaces were reconstructed using the *recon-all* command in FreeSurfer (Dale, Fischl, & Sereno, 1999), and the brain mask previously estimated was refined with a custom variation of the method to reconcile ANTs-derived and FreeSurfer-derived segmentations of the cortical gray matter of Mindboggle (Klein et al., 2017). Grayordinate *dscalar* files (Glasser et al., 2013) containing 91k samples were generated using the highest-resolution *fsaverage* as an intermediate standardized surface space. Volume-based spatial normalization to MNI152NLin6Asym standard space (Evans, Janke, Collins, & Baillet, 2012) was performed through nonlinear registration with *antsRegistration* (ANTs) and accessed with TemplateFlow (Ciric et al., 2022), using brain-extracted versions of both T1w reference and the T1w template.

rs-BOLD-fMRI data were preprocessed as follows. First, a reference volume and its skull-stripped version were generated using a custom methodology of *fMRIPrep*. Head-motion parameters with respect to the BOLD reference (transformation matrices, and 6 corresponding rotation and translation parameters) were estimated before spatiotemporal filtering using *mcflirt* (FSL, Jenkinson *et al.,* 2002) (Jenkinson, Bannister, Brady, & Smith, 2002). The BOLD time-series were resampled onto their original, native space by applying the transforms to correct for head motion. These resampled BOLD time-series are termed ‘preprocessed BOLD in original space’, or just ‘preprocessed BOLD’. The BOLD reference was then co-registered to the T1w reference using *bbregister* (FreeSurfer) which implements boundary-based registration (Greve & Fischl, 2009), configured with 6 degrees of freedom. Several confounding time-series were calculated based on the preprocessed BOLD: framewise displacement (FD), DVARS and three region-wise global signals. FD was computed using two formulations following Power (Power et al., 2014) (absolute sum of relative motions) and Jenkinson (Jenkinson et al., 2002) (relative root mean square displacement between affines). FD and DVARS were calculated for each functional run, using their implementations in *Nipype*. The three global signals were extracted within the CSF, the WM, and the whole-brain masks. Additionally, a set of physiological regressors were extracted to allow for component-based noise correction (Behzadi, Restom, Liau, & Liu, 2007) (CompCor). Principal components were estimated after high-pass filtering the preprocessed BOLD time-series (using a discrete cosine filter with 128s cut-off) for the two CompCor variants: temporal (tCompCor) and anatomical (aCompCor). tCompCor components were then calculated from the top 2% variable voxels within the brain mask. For aCompCor, three probabilistic masks (CSF, WM and combined CSF+WM) were generated in anatomical space. The implementation differed from that of Behzadi *et al*. in that instead of eroding the masks by 2 pixels on BOLD space (Behzadi et al., 2007), a mask of pixels that likely contain a volume fraction of GM was subtracted from the *aCompCor* masks. This mask was obtained by dilating a GM mask extracted from the FreeSurfer’s *aseg* segmentation, and ensures that components are not extracted from voxels containing only a minimal fraction of GM. Finally, these masks were resampled into BOLD space and binarized by thresholding at 0.99 (as in the original implementation (Behzadi et al., 2007)). Components were also calculated separately within the WM and CSF masks. For each *CompCor* decomposition, the k components with the largest singular values were retained, such that the retained components’ time series are sufficient to explain 50% of variance across the nuisance mask (CSF, WM, combined, or temporal). The remaining components were discarded. The head-motion estimates calculated in the correction step were also placed within the corresponding confounds file. The confound time series derived from head motion estimates and global signals were expanded with the inclusion of temporal derivatives and quadratic terms for each (Satterthwaite et al., 2013). Frames that exceeded 0.5 mm FD or 1.5 standardized DVARS were annotated as motion outliers. Additional nuisance timeseries were calculated by principal components analysis of the signal found within a thin band (crown) of voxels around the edge of the brain (Patriat, Reynolds, & Birn, 2017). The BOLD time-series were resampled into standard space, generating a preprocessed BOLD run in MNI152NLin6Asym space. First, a reference volume and its skull-stripped version were generated using a custom methodology of *fMRIPrep*. The BOLD time-series were resampled onto two surfaces (FreeSurfer reconstruction nomenclature): *fsaverage5* and *fsaverage6*. The BOLD time-series were resampled onto the left/right-symmetric template fsLR (Glasser et al., 2013). Grayordinates files containing 91k samples were also generated using the highest-resolution *fsaverage* as intermediate standardized surface space. All resamplings were performed with a single interpolation step by composing all the pertinent transformations (i.e. head-motion transform matrices, susceptibility distortion correction when available, and co-registrations to anatomical and output spaces). Gridded (volumetric) resamplings were performed using *antsApplyTransforms* (ANTs), configured with Lanczos interpolation to minimize the smoothing effects of other kernels (Lanczos, 2006). Non-gridded (surface) resamplings were performed using mri_vol2surf (FreeSurfer).

The eXtensible Connectivity Pipeline- DCAN (*XCP-D*) (Ciric et al., 2018; Satterthwaite et al., 2013) was used to post-process the outputs of *fMRIPrep*, which was also built with *Nipype* 1.8.6. Native-space T1w images were transformed to MNI152NLin6Asym space at 1 mm^3^ resolution. Each pre-processed rs-BOLD-fMRI dataset was post-processed as follows. In order to identify high-motion outlier volumes, Power FD was calculated with a head radius of 40.0 mm and volumes with FD > 0.3 mm were flagged as high-motion outliers for later censoring (Power et al., 2014). In accordance with the ‘36P’ strategy, a total of 36 nuisance regressors were selected from the preprocessing confounds, including 6 motion parameters, mean global, white matter and cerebrospinal fluid signals with their temporal derivatives, and quadratic expansion of 6 motion parameters, tissue signals and their temporal derivatives (Ciric et al., 2017; Satterthwaite et al., 2013). Finally, linear trend and intercept terms were added to the regressors prior to denoising. The BOLD data were converted to NIfTI format, despiked with AFNI’s 3dDespike (Cox, 1996; Cox & Hyde, 1997), and converted back to CIFTI format. Nuisance regressors were regressed from the BOLD data using linear regression, implemented in *Nilearn*. Any volumes censored earlier in the workflow were interpolated in the residual time series produced by the regression, then the interpolated timeseries were band-pass filtered using a second-order Butterworth filter to retain signals between 0.01-0.08 Hz. The filtered interpolated time series were re-censored to remove high-motion outlier volumes. The denoised BOLD was smoothed using Connectome Workbench with a Gaussian kernel (FWHM 6.0 mm) (Marcus et al., 2011). Processed functional timeseries were extracted from residual BOLD data using Connectome Workbench for the Schaefer 100 atlas (Schaefer et al., 2018). Pair-wise functional connectivity between all regions was computed for each atlas, operationalized as the Pearson’s correlation of each parcel’s unsmoothed timeseries with the Connectome Workbench. In cases of partial coverage, uncovered vertices (values of all zero or NaN) were either ignored (when the parcel had ≥ 50% coverage) or set to zero (when it had < 50% coverage).

# Structural (DTI) data preprocessing

DTI data were preprocessed and reconstruction using *QSIPrep* 0.19.1 (Cieslak et al., 2021), which is based on *Nipype* 1.8.6. The T1w image was corrected for INU and used as an anatomical reference image throughout the workflow. This was reoriented into AC-PC alignment via a 6-DOF transform extracted from a full Affine registration to the MNI template. A full nonlinear registration to the template from AC-PC space was estimated via symmetric nonlinear registration (SyN) using *antsRegistration* (ANTs). Brain extraction was performed using *SynthStrip* (Hoopes, Mora, Dalca, Fischl, & Hoffmann, 2022) followed by automated segmentation using *SynthSeg* (Billot et al., 2023) from FreeSurfer.

Any images with b˂100 s/mm^2^ were treated as a b=0 image. MP-PCA denoising as implemented in MRtrix3’s *dwidenoise* (Veraart et al., 2016) was applied with a 5-voxel window. The mean intensity of the DWI series was then adjusted so the mean intensity of all the b=0 images matched across each separate DWI scanning sequence. B1 field inhomogeneity was corrected using *dwibiascorrect* from MRtrix3 with the N4 algorithm (Tustison et al., 2010) after corrected images were resampled.

FSL’s *eddy* was used for head motion correction and eddy current correction (Andersson & Sotiropoulos, 2016), configured with q-space smoothing factor 10, a total of 5 iterations, using 1000 voxels to estimate hyperparameters. A linear first level model and a linear second level model were used to characterize eddy current-related spatial distortion. q-space coordinates were forcefully assigned to shells. The effects of field offset displacement and subject movement were separated as far as possible. Shells were aligned post-eddy. *eddy’s* outlier replacement was run (Andersson, Graham, Zsoldos, & Sotiropoulos, 2016). Data were grouped by slice, including only slices with ≥250 intracerebral voxels. Groups deviating by >4 standard deviations from the prediction had their data replaced with imputed values. Final interpolation was performed using the *jac* method.

Several confounding time-series were calculated based on the preprocessed DWI: FD as defined by Power (Power et al., 2014); head-motion estimates calculated in the correction step were placed within the corresponding confounds file; slice-wise cross-correlation. The DWI time-series were resampled to ACPC, generating a preprocessed DWI run in ACPC space with 1.5mm isotropic voxels.

T1w-based spatial normalization calculated during preprocessing was used to map atlases from template space into alignment with DWIs. Multi-tissue fiber response functions were estimated using the *dhollander* algorithm. Fiber Orientation Distributions (FODs) were estimated via constrained spherical deconvolution (CSD) (Tournier, Calamante, Gadian, & Connelly, 2004; Tournier et al., 2008) using an unsupervised multi-tissue method (Dhollander, Raffelt, & Connelly, 2016). A single-shell-optimized multi-tissue CSD was performed using MRtrix3Tissue (<https://3Tissue.github.io>), a fork of MRtrix3 (Tournier et al., 2019) FODs were intensity-normalized using *mtnormalize* (Raffelt et al., 2017).

# Gene expression data processing

Regional microarray expression data obtained from 6 post-mortem brains (1 female, ages 24-57 y, mean 43±13 y) were provided by the Allen Human Brain Atlas (AHBA, <https://human.brain-map.org>) (M. J. Hawrylycz et al., 2012). Data were processed with the *abagen* toolbox (v. 0.1.4+15.gdc4a007; <https://github.com/rmarkello/abagen>) (Markello et al., 2021) using a 152-region atlas in MNI space. First, microarray probes were reannotated using data provided by Arnatkeviciute *et al*. (Arnatkeviciute, Fulcher, & Fornito, 2019); probes not matched to a valid Entrez ID were discarded. Probes were filtered based on their expression intensity relative to background noise (Quackenbush, 2002), discarding probes with intensity less than background in ≥50% of samples across donors, yielding 31,569 probes. When multiple probes indexed expression of the same gene, we used the probe with the most consistent pattern of regional variation across donors (M. Hawrylycz et al., 2015). Regions correspond to the structural designations provided in the ontology from the AHBA.

The MNI coordinates of tissue samples were updated to those generated via non-linear registration using Advanced Normalization Tools (ANT; <https://github.com/chrisfilo/alleninf>). Samples were assigned to brain regions in the atlas if their MNI coordinates were within 2 mm of a given parcel. To minimise misassignment, sample-to-region matching was constrained by hemisphere and gross structural divisions (cortex, subcortex/brainstem, and cerebellum), such that e.g. a sample in the left cortex could only be assigned to an atlas parcel in the left cortex (Arnatkeviciute et al., 2019). Tissue samples not assigned to a brain region in the atlas were discarded. Inter-subject variation was addressed by normalizing tissue sample expression values across genes using a robust sigmoid function (Fulcher, Little, & Jones, 2013).

Gene expression values were normalized across tissue samples using an identical procedure. Normalization was performed separately for samples in distinct structural classes (i.e. cortex, subcortex/brainstem, cerebellum). Samples assigned to the same region were averaged separately for each donor and then across donors, yielding a regional expression matrix with 152 rows (corresponding to brain regions) and 15,677 columns (corresponding to retained genes).

**Table S1. Definition of the 152 regions of interest (ROI)**

| ROI label | Atlas source | ROI label | Atlas source |
| --- | --- | --- | --- |
| LH_Vis_1 | Schaefer100 | RH_Vis_1 | Schaefer100 |
| LH_Vis_2 | Schaefer100 | RH_Vis_2 | Schaefer100 |
| LH_Vis_3 | Schaefer100 | RH_Vis_3 | Schaefer100 |
| LH_Vis_4 | Schaefer100 | RH_Vis_4 | Schaefer100 |
| LH_Vis_5 | Schaefer100 | RH_Vis_5 | Schaefer100 |
| LH_Vis_6 | Schaefer100 | RH_Vis_6 | Schaefer100 |
| LH_Vis_7 | Schaefer100 | RH_Vis_7 | Schaefer100 |
| LH_Vis_8 | Schaefer100 | RH_Vis_8 | Schaefer100 |
| LH_Vis_9 | Schaefer100 | RH_SomMot_1 | Schaefer100 |
| LH_SomMot_1 | Schaefer100 | RH_SomMot_2 | Schaefer100 |
| LH_SomMot_2 | Schaefer100 | RH_SomMot_3 | Schaefer100 |
| LH_SomMot_3 | Schaefer100 | RH_SomMot_4 | Schaefer100 |
| LH_SomMot_4 | Schaefer100 | RH_SomMot_5 | Schaefer100 |
| LH_SomMot_5 | Schaefer100 | RH_SomMot_6 | Schaefer100 |
| LH_SomMot_6 | Schaefer100 | RH_SomMot_7 | Schaefer100 |
| LH_DorsAttn_Post_1 | Schaefer100 | RH_SomMot_8 | Schaefer100 |
| LH_DorsAttn_Post_2 | Schaefer100 | RH_DorsAttn_Post_1 | Schaefer100 |
| LH_DorsAttn_Post_3 | Schaefer100 | RH_DorsAttn_Post_2 | Schaefer100 |
| LH_DorsAttn_Post_4 | Schaefer100 | RH_DorsAttn_Post_3 | Schaefer100 |
| LH_DorsAttn_Post_5 | Schaefer100 | RH_DorsAttn_Post_4 | Schaefer100 |
| LH_DorsAttn_Post_6 | Schaefer100 | RH_DorsAttn_Post_5 | Schaefer100 |
| LH_DorsAttn_PrCv_1 | Schaefer100 | RH_DorsAttn_PrCv_1 | Schaefer100 |
| LH_DorsAttn_FEF_1 | Schaefer100 | RH_DorsAttn_FEF_1 | Schaefer100 |
| LH_SalVentAttn_ParOper_1 | Schaefer100 | RH_SalVentAttn_TempOccPar_1 | Schaefer100 |
| LH_SalVentAttn_FrOperIns_1 | Schaefer100 | RH_SalVentAttn_TempOccPar_2 | Schaefer100 |
| LH_SalVentAttn_FrOperIns_2 | Schaefer100 | RH_SalVentAttn_FrOperIns_1 | Schaefer100 |
| LH_SalVentAttn_PFCl_1 | Schaefer100 | RH_SalVentAttn_Med_1 | Schaefer100 |
| LH_SalVentAttn_Med_1 | Schaefer100 | RH_SalVentAttn_Med_2 | Schaefer100 |
| LH_SalVentAttn_Med_2 | Schaefer100 | RH_Limbic_OFC_1 | Schaefer100 |
| LH_SalVentAttn_Med_3 | Schaefer100 | RH_Limbic_TempPole_1 | Schaefer100 |
| LH_Limbic_OFC_1 | Schaefer100 | RH_Cont_Par_1 | Schaefer100 |
| LH_Limbic_TempPole_1 | Schaefer100 | RH_Cont_Par_2 | Schaefer100 |
| LH_Limbic_TempPole_2 | Schaefer100 | RH_Cont_PFCl_1 | Schaefer100 |
| LH_Cont_Par_1 | Schaefer100 | RH_Cont_PFCl_2 | Schaefer100 |
| LH_Cont_PFCl_1 | Schaefer100 | RH_Cont_PFCl_3 | Schaefer100 |
| LH_Cont_pCun_1 | Schaefer100 | RH_Cont_PFCl_4 | Schaefer100 |
| LH_Cont_Cing_1 | Schaefer100 | RH_Cont_Cing_1 | Schaefer100 |
| LH_Default_Temp_1 | Schaefer100 | RH_Cont_PFCmp_1 | Schaefer100 |
| LH_Default_Temp_2 | Schaefer100 | RH_Cont_pCun_1 | Schaefer100 |
| LH_Default_Par_1 | Schaefer100 | RH_Default_Par_1 | Schaefer100 |
| LH_Default_Par_2 | Schaefer100 | RH_Default_Temp_1 | Schaefer100 |
| LH_Default_PFC_1 | Schaefer100 | RH_Default_Temp_2 | Schaefer100 |
| LH_Default_PFC_2 | Schaefer100 | RH_Default_Temp_3 | Schaefer100 |
| LH_Default_PFC_3 | Schaefer100 | RH_Default_PFCv_1 | Schaefer100 |
| LH_Default_PFC_4 | Schaefer100 | RH_Default_PFCv_2 | Schaefer100 |
| LH_Default_PFC_5 | Schaefer100 | RH_Default_PFCdPFCm_1 | Schaefer100 |
| LH_Default_PFC_6 | Schaefer100 | RH_Default_PFCdPFCm_2 | Schaefer100 |
| LH_Default_PFC_7 | Schaefer100 | RH_Default_PFCdPFCm_3 | Schaefer100 |
| LH_Default_pCunPCC_1 | Schaefer100 | RH_Default_pCunPCC_1 | Schaefer100 |
| LH_Default_pCunPCC_2 | Schaefer100 | RH_Default_pCunPCC_2 | Schaefer100 |
| LH-Pu | CIT168Subcortical | RH-Pu | CIT168Subcortical |
| LH-Ca | CIT168Subcortical | RH-Ca | CIT168Subcortical |
| LH-NAC | CIT168Subcortical | RH-NAC | CIT168Subcortical |
| LH-EXA | CIT168Subcortical | RH-EXA | CIT168Subcortical |
| LH-GPe | CIT168Subcortical | RH-GPe | CIT168Subcortical |
| LH-GPi | CIT168Subcortical | RH-GPi | CIT168Subcortical |
| LH-SNc_PBP_VTA | CIT168Subcortical | RH-SNc_PBP_VTA | CIT168Subcortical |
| LH-RN | CIT168Subcortical | RH-RN | CIT168Subcortical |
| LH-SNr | CIT168Subcortical | RH-SNr | CIT168Subcortical |
| LH-VeP | CIT168Subcortical | RH-VeP | CIT168Subcortical |
| LH-HN | CIT168Subcortical | RH-HN | CIT168Subcortical |
| LH-HTH | CIT168Subcortical | RH-HTH | CIT168Subcortical |
| LH-MN | CIT168Subcortical | RH-MN | CIT168Subcortical |
| LH-STH | CIT168Subcortical | RH-STH | CIT168Subcortical |
| LH-Pulvinar | ThalamusHCP | RH-Pulvinar | ThalamusHCP |
| LH-Anterior | ThalamusHCP | RH-Anterior | ThalamusHCP |
| LH-Medio_Dorsal | ThalamusHCP | RH-Medio_Dorsal | ThalamusHCP |
| LH-Ventral_Latero_Dorsal | ThalamusHCP | RH-Ventral_Latero_Dorsal | ThalamusHCP |
| LH-Central_Lateral-Lateral_Posterior-Medial_Pulvinar | ThalamusHCP | RH-Central_Lateral-Lateral_Posterior-Medial_Pulvinar | ThalamusHCP |
| LH-Ventral_Anterior | ThalamusHCP | RH-Ventral_Anterior | ThalamusHCP |
| LH-Ventral_Latero_Ventral | ThalamusHCP | RH-Ventral_Latero_Ventral | ThalamusHCP |
| Cerebellar_Region1 | Cerebellum | Cerebellar_Region6 | Cerebellum |
| Cerebellar_Region2 | Cerebellum | Cerebellar_Region7 | Cerebellum |
| Cerebellar_Region3 | Cerebellum | Cerebellar_Region8 | Cerebellum |
| Cerebellar_Region4 | Cerebellum | Cerebellar_Region9 | Cerebellum |
| Cerebellar_Region5 | Cerebellum | Cerebellar_Region10 | Cerebellum |

**Table S2. Spatial colocalization maps**

| Map | Description | Source |
| --- | --- | --- |
| CBF | ASL_MRI_Cerebral blood flow | Holiga *et al*., 2018 |
| GABAa | GABA_Flumazenil_HC16 | Nørgaard *et al*., 2021 |
| GABAa5 | GABA_RO154513_HC10 | Lukow *et al*., 2022 |
| D1 | Dopamine_SCH23390_HC13 | Kaller *et al*., 2017 |
| D2 | Dopamine_Fallypride_HC49 | Jaworska *et al*., 2020 |
| DAT | Dopamine_FP-CIT_HC174 | Dukart *et al*., 2018 |
| NAT | Norepinephrine_MRB | Hesse *et al*., 2017 |
| SERT | Serotonin_DASB_HC100 | Beliveau *et al*., 2017 |
| 5HT1a | Serotonin_WAY-100635_HC36 | Savli *et al*., 2012 |
| 5HT1b | Serotonin_P943_HC22 | Savli *et al*., 2012 |
| 5HT2a | Serotonin_ALT_HC19 | Savli *et al*., 2012 |
| 5HT4 | Serotonin_SB20_HC59 | Beliveau *et al*., 2017 |
| 5HT6 | Serotonin_GSK215083_HC30 | Radhakrishnan *et al.*, 2018 |
| MU | Carfentanil_HC39 | Turtonen *et al*., 2021 |
| Kappa Opiod | LY2795050_HC64 | Shokri-Kojori *et al.*, 2022 |
| CMRglu | FDG_HC20 | Castrillon *et al.*, 2023 |
| mGluR5 | Glutamate_ABP_HC73 | Smart *et al*., 2019 |
| NMDA | Glutamate_GE179_HC29 | Galovic *et al*., 2021 |
| CB1 | Cannabinoid_omar_HC22 | Laurikainen *et al*., 2019 |
| MOR | Opioid_Carfentanil_HC204 | Kantonen *et al.*, 2020 |
| VAChT | Acetylcholine_feobv_HC4 | Hansen *et al*., 2022 |
| M1 | Acetylcholine_LSN3172176_HC24 | Naganawa *et al*., 2021 |
| A4B2 | Acetylcholine_flubatine_HC30 | Hillmer *et al*., 2016 |
| COX1 | PS13_HC11 | Kim *et al*., 2020 |
| HDAC | Martinostat_HC8 | Wey *et al*., 2016 |
| SV2A | UCBJ_HC10 | Finnema *et al*., 2018 |
| VMAT2 | DTBZ_HC76 | Larsen *et al*., 2020 |
| Neuronal cell | Individual mRNA samples from AHBA | Lake *et al*., 2016 |
| Glial cell | Individual mRNA samples from AHBA | Darmanis *et al*., 2015 |
| Mitochondria | Molecular energetic landscape | Mosharov *et al*., 2025 |

**Table S3. Network level between-group differences in structure-function coupling**

| Network | PTSD | TENC | Mean Difference | *p* |
| --- | --- | --- | --- | --- |
| VN | 0.265 (0.069) | 0.285 (0.069) | -0.020 | 0.328 |
| SMN | 0.136 (0.038) | 0.153 (0.033) | -0.016 | 0.124 |
| DAN | 0.175 (0.044) | 0.181 (0.040) | -0.006 | 0.658 |
| VAN | 0.160 (0.037) | 0.152 (0.036) | 0.008 | 0.380 |
| LN | 0.175 (0.051) | 0.175 (0.053) | 0.001 | 0.991 |
| FPN | 0.158 (0.029) | 0.149 (0.027) | 0.009 | 0.278 |
| DMN | 0.138 (0.023) | 0.138 (0.026) | 0.000 | 0.991 |
| SUB | 0.173 (0.042) | 0.185 (0.045) | -0.012 | 0.328 |
| CE | 0.423 (0.121) | 0.433 (0.134) | -0.010 | 0.853 |

Note: *p* corrected by False Discovery Rate (no *p* < 0.05 survived correction). Abbreviations: CE, cerebellum region; DAN, dorsal attention network; DMN, default mode network; FPN, frontoparietal control network; LN, limbic network; PTSD, post-traumatic stress disorder; SMN, sensorimotor network; SUB, subcortical area; TENC, trauma-exposed non-PTSD controls; VAN, ventral attention network; VN, visual network.

**Table S4. Results of enrichment analysis**

| **Category** | **ID** | **Description** | **Gene Ratio** | **-log_10_(q)** | **p (FDR adjusted)** |
| --- | --- | --- | --- | --- | --- |
| **PLS2+ genes** | | | | | |
| GO: BP | GO:0001659 | temperature homeostasis | 0.030 | 3.807 | 0.0002 |
| GO: BP | GO:0120161 | regulation of cold-induced thermogenesis | 0.024 | 2.762 | 0.0019 |
| GO: BP | GO:1990845 | adaptive thermogenesis | 0.025 | 2.534 | 0.0032 |
| GO: BP | GO:0051960 | regulation of nervous system development | 0.046 | 1.806 | 0.0172 |
| GO: BP | GO:0007264 | small GTPase-mediated signal transduction | 0.048 | 1.784 | 0.0181 |
| GO:MF | GO:0005201 | extracellular matrix structural constituent | 0.022 | 1.584 | 0.0283 |
| GO:MF | GO:0030695 | GTPase regulator activity | 0.046 | 1.584 | 0.0283 |
| GO:MF | GO:0060589 | nucleoside-triphosphatase regulator activity | 0.046 | 1.584 | 0.0283 |
| GO:MF | GO:1901681 | sulfur compound binding | 0.030 | 1.438 | 0.0396 |
| GO:CC | GO:0062023 | collagen-containing extracellular matrix | 0.043 | 2.422 | 6.96E-06 |
| GO:CC | GO:0005912 | adherens junction | 0.024 | 2.371 | 1.57E-05 |
| GO:CC | GO:0005604 | basement membrane | 0.014 | 1.320 | 0.000264 |
| KEGG | hsa04360 | Axon guidance | 0.042 | 1.710 | 0.0231 |
| KEGG | hsa04082 | Neuroactive ligand signaling | 0.044 | 1.710 | 0.0231 |
| KEGG | hsa04916 | Melanogenesis | 0.028 | 1.710 | 0.0231 |
| **PLS2- genes** | | | | | |
| GO: BP | GO:0007272 | ensheathment of neurons | 0.028 | 3.819 | 0.0002 |
| GO: BP | GO:0051960 | regulation of nervous system development | 0.052 | 3.311 | 0.0005 |
| GO: BP | GO:0097107 | postsynaptic density assembly | 0.011 | 3.302 | 0.0005 |
| GO: BP | GO:0010975 | regulation of neuron projection development | 0.049 | 2.742 | 0.0019 |
| GO: BP | GO:0007416 | synapse assembly | 0.035 | 2.739 | 0.0019 |
| GO:MF | GO:0001540 | amyloid-beta binding | 0.015 | 1.456 | 0.0357 |
| GO:MF | GO:0005230 | extracellular ligand-gated monoatomic ion channel activity | 0.014 | 1.456 | 0.0357 |
| GO:CC | GO:0097060 | synaptic membrane | 0.060 | 7.812 | 1.67E-08 |
| GO:CC | GO:0045211 | postsynaptic membrane | 0.044 | 5.967 | 1.17E-06 |
| GO:CC | GO:0099634 | postsynaptic specialization membrane | 0.027 | 5.277 | 5.74E-06 |
| KEGG | hsa04723 | Retrograde endocannabinoid signaling | 0.050 | 3.167 | 0.0008 |
| KEGG | hsa05022 | Pathways of neurodegeneration - multiple diseases | 0.098 | 2.195 | 0.0075 |
| KEGG | hsa04082 | Neuroactive ligand signaling | 0.050 | 1.857 | 0.0163 |

Abbreviations: BP, biological processes; CC, cellular components; GO, Gene Ontology; KEGG, Kyoto Encyclopedia of Genes and Genomes; MF, molecular functions; PLS2, the second component of the PLS regression.


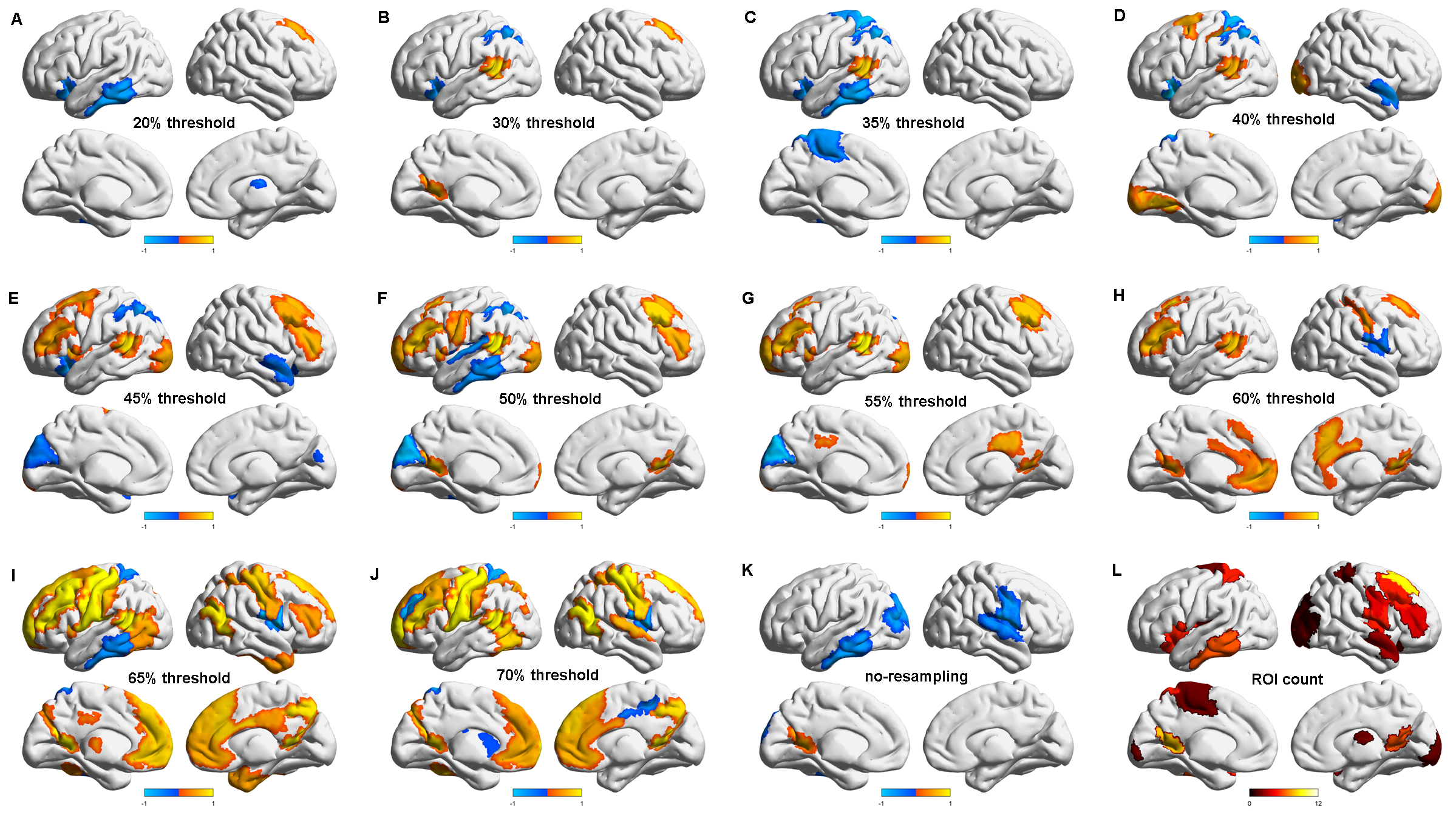


# Figure S1. Group-wise differences using different thresholds (Panels A-J) and normalization (Panel K), and statistically significant regions common to all tested parameters (Panel L)

To evaluate the influence on our results of thresholding and normalization, we repeated structural connectivity network (SCN) construction using thresholds from 20-70% with a 5% step and using non-resampled SCN. For each SCN, we computed SFC and compared it between PTSD and trauma-exposed non-PTSD control groups exactly as in the main text. Cohen’s *d* calculated for each region of interest (ROI) was the effect size measure for ROI-level inter-group SFC difference.

Panels A-K show regions with statistically significant between-group SFC differences (colour-coded for Cohen’s *d*) with the different parameter settings noted. Panel L summarises the (colour-coded) count of significant ROIs identified in the main text that remained statistically significant across all tested parameter settings: a higher count indicates lower sensitivity to parameter variations.


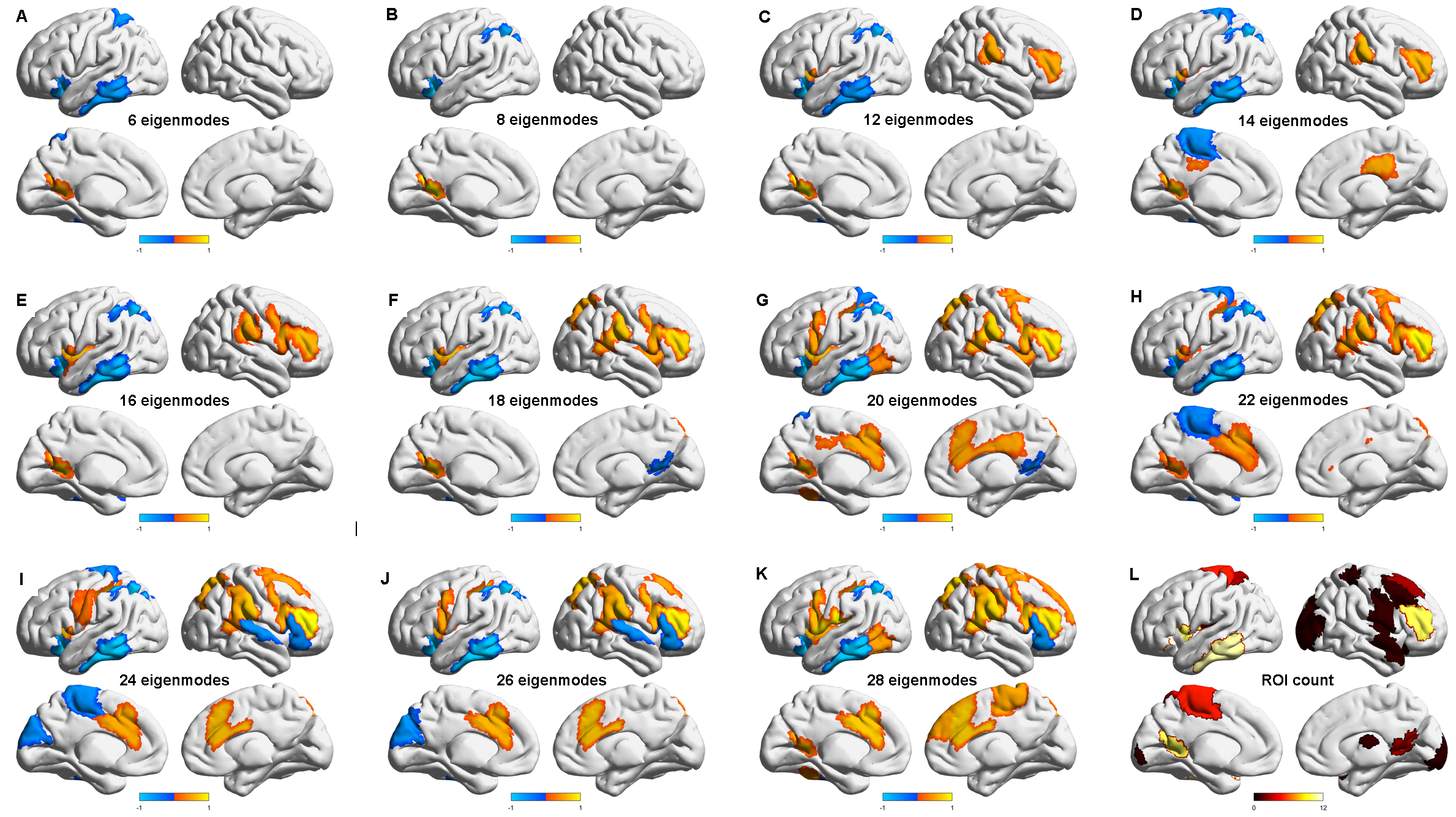


**Figure S2. Group-wise differences using different eigenmodes (Panel A-K), and statistically significant regions common to all tested parameters (Panel L)**

To evaluate the influence on our results of the chosen number of eigenmodes we repeated the SFC calculation varying the number of eigenmodes from 6-28 in steps of 2. SFC was compared between PTSD and trauma-exposed non-PTSD control groups for each eigenmode set exactly as in the main text. Cohen’s *d* calculated for each region of interest (ROI) was the effect size measure for ROI-level inter-group SFC difference.

Panels A-K show regions with statistically significant between-group differences in SFC (colour-coded for Cohen’s *d*) with the different eigenmode numbers noted. Panel L summarises the (colour-coded) count of significant ROIs identified in the main text that remained statistically significant across all tested eigenmode numbers: a higher count indicates lower sensitivity to eigenmode number variations.


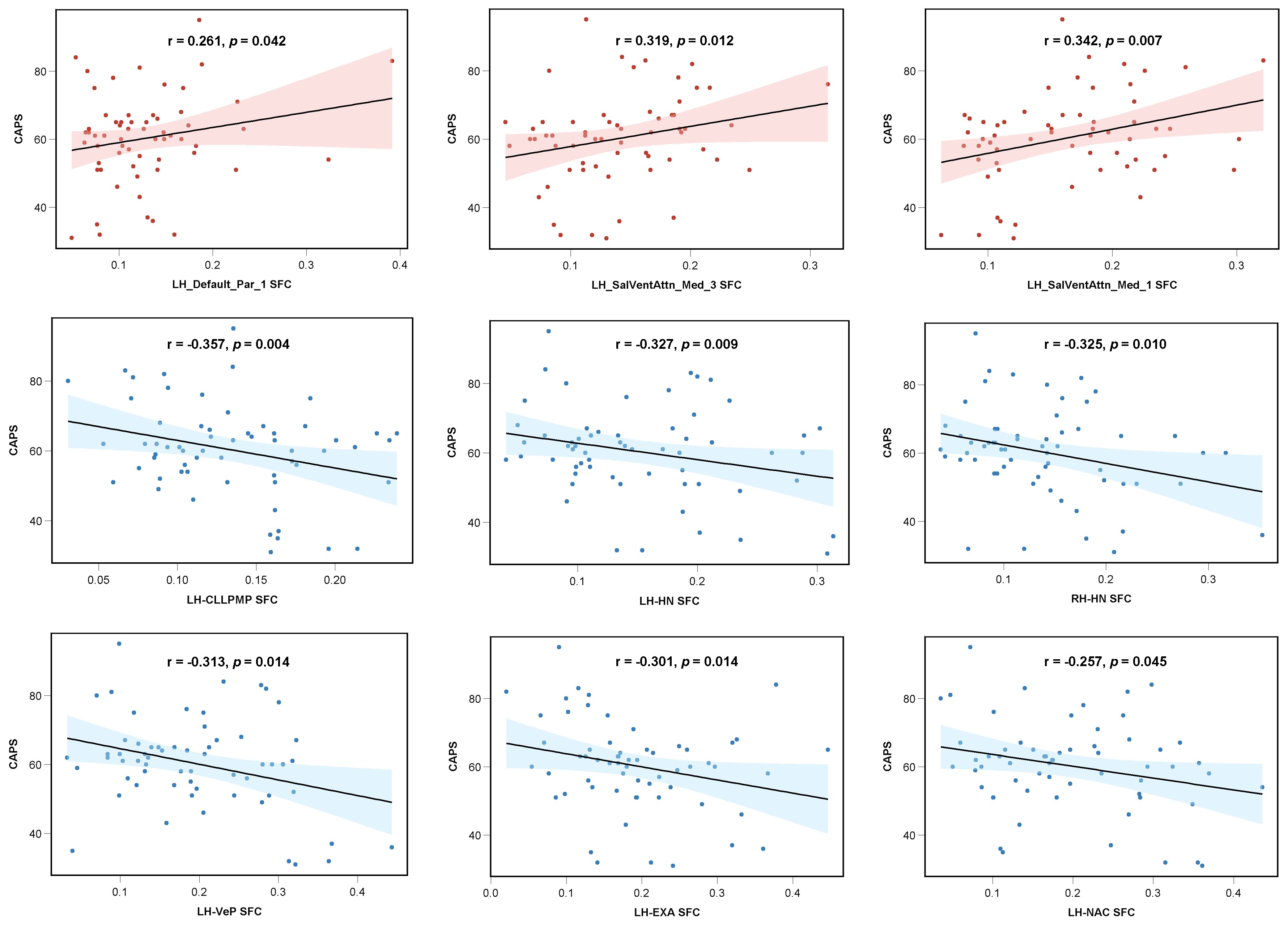


**Figure S3. Results of partial correlation analysis in non-significant brain regions**

Scatterplots showing associations (not significant after FDR correction) in PTSD patients between the Clinician-Administered

PTSD Scale score (CAPS) and structure-function coupling (SFC) in brain regions which show no significant between-group differences.

**References**

Andersson, J. L. R., Graham, M. S., Zsoldos, E., & Sotiropoulos, S. N. (2016). Incorporating outlier detection and replacement into a non-parametric framework for movement and distortion correction of diffusion MR images. *NeuroImage*, *141*, 556–572. https://doi.org/10.1016/j.neuroimage.2016.06.058

Andersson, J. L. R., & Sotiropoulos, S. N. (2016). An integrated approach to correction for off-resonance effects and subject movement in diffusion MR imaging. *NeuroImage*, *125*, 1063–1078. https://doi.org/10.1016/j.neuroimage.2015.10.019

Arnatkeviciute, A., Fulcher, B. D., & Fornito, A. (2019). A practical guide to linking brain-wide gene expression and neuroimaging data. *NeuroImage*, *189*, 353–367. https://doi.org/10.1016/j.neuroimage.2019.01.011

Avants, B. B., Epstein, C. L., Grossman, M., & Gee, J. C. (2008). Symmetric diffeomorphic image registration with cross-correlation: Evaluating automated labeling of elderly and neurodegenerative brain. *Medical Image Analysis*, *12*(1), 26–41. https://doi.org/10.1016/j.media.2007.06.004

Behzadi, Y., Restom, K., Liau, J., & Liu, T. T. (2007). A component based noise correction method (CompCor) for BOLD and perfusion based fMRI. *NeuroImage*, *37*(1), 90–101. https://doi.org/10.1016/j.neuroimage.2007.04.042

Beliveau, V., Ganz, M., Feng, L., Ozenne, B., Højgaard, L., Fisher, P. M., … Knudsen, G. M. (2017). A High-Resolution In Vivo Atlas of the Human Brain’s Serotonin System. *The Journal of Neuroscience*, *37*(1), 120–128. https://doi.org/10.1523/JNEUROSCI.2830-16.2016

Billot, B., Greve, D. N., Puonti, O., Thielscher, A., Van Leemput, K., Fischl, B., … ADNI. (2023). SynthSeg: Segmentation of brain MRI scans of any contrast and resolution without retraining. *Medical Image Analysis*, *86*, 102789. https://doi.org/10.1016/j.media.2023.102789

Blake, D. D., Weathers, F. W., Nagy, L. M., Kaloupek, D. G., Gusman, F. D., Charney, D. S., & Keane, T. M. (1995). The development of a Clinician-Administered PTSD Scale. *Journal of Traumatic Stress*, *8*(1), 75–90. https://doi.org/10.1007/BF02105408

Castrillon, G., Epp, S., Bose, A., Fraticelli, L., Hechler, A., Belenya, R., … Riedl, V. (2023). An energy costly architecture of neuromodulators for human brain evolution and cognition. *Science Advances*, *9*(50), eadi7632. https://doi.org/10.1126/sciadv.adi7632

Cieslak, M., Cook, P. A., He, X., Yeh, F.-C., Dhollander, T., Adebimpe, A., … Satterthwaite, T. D. (2021). QSIPrep: An integrative platform for preprocessing and reconstructing diffusion MRI data. *Nature Methods*, *18*(7), 775–778. https://doi.org/10.1038/s41592-021-01185-5

Ciric, R., Rosen, A. F. G., Erus, G., Cieslak, M., Adebimpe, A., Cook, P. A., … Satterthwaite, T. D. (2018). Mitigating head motion artifact in functional connectivity MRI. *Nature Protocols*, *13*(12), 2801–2826. https://doi.org/10.1038/s41596-018-0065-y

Ciric, R., Thompson, W. H., Lorenz, R., Goncalves, M., MacNicol, E. E., Markiewicz, C. J., … Esteban, O. (2022). TemplateFlow: FAIR-sharing of multi-scale, multi-species brain models. *Nature Methods*, *19*(12), 1568–1571. https://doi.org/10.1038/s41592-022-01681-2

Ciric, R., Wolf, D. H., Power, J. D., Roalf, D. R., Baum, G. L., Ruparel, K., … Satterthwaite, T. D. (2017). Benchmarking of participant-level confound regression strategies for the control of motion artifact in studies of functional connectivity. *NeuroImage*, *154*, 174–187. https://doi.org/10.1016/j.neuroimage.2017.03.020

Cox, R. W. (1996). AFNI: Software for analysis and visualization of functional magnetic resonance neuroimages. *Computers and Biomedical Research*, *29*(3), 162–173. https://doi.org/10.1006/cbmr.1996.0014

Cox, R. W., & Hyde, J. S. (1997). Software tools for analysis and visualization of fMRI data. *NMR in Biomedicine*, *10*(4–5), 171–178. https://doi.org/10.1002/(sici)1099-1492(199706/08)10:4/5<171::aid-nbm453>3.0.co;2-l

Dale, A. M., Fischl, B., & Sereno, M. I. (1999). Cortical surface-based analysis. I. Segmentation and surface reconstruction. *NeuroImage*, *9*(2), 179–194. https://doi.org/10.1006/nimg.1998.0395

Darmanis, S., Sloan, S. A., Zhang, Y., Enge, M., Caneda, C., Shuer, L. M., … Quake, S. R. (2015). A survey of human brain transcriptome diversity at the single cell level. *Proceedings of the National Academy of Sciences of the United States of America*, *112*(23), 7285–7290. https://doi.org/10.1073/pnas.1507125112

Dhollander, T., Raffelt, D., & Connelly, A. (2016). Unsupervised 3-tissue response function estimation from single-shell or multi-shell diffusion MR data without a co-registered T1 image. ISMRM Workshop on Breaking the Barriers of Diffusion MRI.

Dukart, J., Holiga, Š., Chatham, C., Hawkins, P., Forsyth, A., McMillan, R., … Sambataro, F. (2018). Cerebral blood flow predicts differential neurotransmitter activity. *Scientific Reports*, *8*(1), 4074. https://doi.org/10.1038/s41598-018-22444-0

Esteban, O., Markiewicz, C. J., Blair, R. W., Moodie, C. A., Isik, A. I., Erramuzpe, A., … Gorgolewski, K. J. (2019). fMRIPrep: A robust preprocessing pipeline for functional MRI. *Nature Methods*, *16*(1), 111–116. https://doi.org/10.1038/s41592-018-0235-4

Evans, A. C., Janke, A. L., Collins, D. L., & Baillet, S. (2012). Brain templates and atlases. *NeuroImage*, *62*(2), 911–922. https://doi.org/10.1016/j.neuroimage.2012.01.024

Finnema, S. J., Nabulsi, N. B., Mercier, J., Lin, S.-F., Chen, M.-K., Matuskey, D., … Carson, R. E. (2018). Kinetic evaluation and test-retest reproducibility of [11C]UCB-J, a novel radioligand for positron emission tomography imaging of synaptic vesicle glycoprotein 2A in humans. *Journal of Cerebral Blood Flow and Metabolism*, *38*(11), 2041–2052. https://doi.org/10.1177/0271678X17724947

First, M. B., Spitzer, R. L., Gibbon, M., & Williams, J. B. (1994). Structured clinical interview for Axis I DSM-IV disorders. New York, NY: Biometrics Research.

Fulcher, B. D., Little, M. A., & Jones, N. S. (2013). Highly comparative time-series analysis: The empirical structure of time series and their methods. *Journal of the Royal Society, Interface*, *10*(83), 20130048. https://doi.org/10.1098/rsif.2013.0048

Galovic, M., Erlandsson, K., Fryer, T. D., Hong, Y. T., Manavaki, R., Sari, H., … NEST investigators. (2021). Validation of a combined image derived input function and venous sampling approach for the quantification of [18F]GE-179 PET binding in the brain. *NeuroImage*, *237*, 118194. https://doi.org/10.1016/j.neuroimage.2021.118194

Glasser, M. F., Sotiropoulos, S. N., Wilson, J. A., Coalson, T. S., Fischl, B., Andersson, J. L., … WU-Minn HCP Consortium. (2013). The minimal preprocessing pipelines for the Human Connectome Project. *NeuroImage*, *80*, 105–124. https://doi.org/10.1016/j.neuroimage.2013.04.127

Gorgolewski, K., Burns, C. D., Madison, C., Clark, D., Halchenko, Y. O., Waskom, M. L., & Ghosh, S. S. (2011). Nipype: A flexible, lightweight and extensible neuroimaging data processing framework in python. *Frontiers in Neuroinformatics*, *5*, 13. https://doi.org/10.3389/fninf.2011.00013

Greve, D. N., & Fischl, B. (2009). Accurate and robust brain image alignment using boundary-based registration. *NeuroImage*, *48*(1), 63–72. https://doi.org/10.1016/j.neuroimage.2009.06.060

Hansen, J. Y., Shafiei, G., Markello, R. D., Smart, K., Cox, S. M. L., Nørgaard, M., … Misic, B. (2022). Mapping neurotransmitter systems to the structural and functional organization of the human neocortex. *Nature Neuroscience*, *25*(11), 1569–1581. https://doi.org/10.1038/s41593-022-01186-3

Hawrylycz, M. J., Lein, E. S., Guillozet-Bongaarts, A. L., Shen, E. H., Ng, L., Miller, J. A., … Jones, A. R. (2012). An anatomically comprehensive atlas of the adult human brain transcriptome. *Nature*, *489*(7416), 391–399. https://doi.org/10.1038/nature11405

Hawrylycz, M., Miller, J. A., Menon, V., Feng, D., Dolbeare, T., Guillozet-Bongaarts, A. L., … Lein, E. (2015). Canonical genetic signatures of the adult human brain. *Nature Neuroscience*, *18*(12), 1832–1844. https://doi.org/10.1038/nn.4171

Hesse, S., Becker, G.-A., Rullmann, M., Bresch, A., Luthardt, J., Hankir, M. K., … Sabri, O. (2017). Central noradrenaline transporter availability in highly obese, non-depressed individuals. *European Journal of Nuclear Medicine and Molecular Imaging*, *44*(6), 1056–1064. https://doi.org/10.1007/s00259-016-3590-3

Hillmer, A. T., Esterlis, I., Gallezot, J. D., Bois, F., Zheng, M. Q., Nabulsi, N., … Cosgrove, K. P. (2016). Imaging of cerebral α4β2* nicotinic acetylcholine receptors with (-)-[(18)F]Flubatine PET: Implementation of bolus plus constant infusion and sensitivity to acetylcholine in human brain. *NeuroImage*, *141*, 71–80. https://doi.org/10.1016/j.neuroimage.2016.07.026

Holiga, Š., Sambataro, F., Luzy, C., Greig, G., Sarkar, N., Renken, R. J., … Dukart, J. (2018). Test-retest reliability of task-based and resting-state blood oxygen level dependence and cerebral blood flow measures. *PloS One*, *13*(11), e0206583. https://doi.org/10.1371/journal.pone.0206583

Hoopes, A., Mora, J. S., Dalca, A. V., Fischl, B., & Hoffmann, M. (2022). SynthStrip: Skull-stripping for any brain image. *NeuroImage*, *260*, 119474. https://doi.org/10.1016/j.neuroimage.2022.119474

Jaworska, N., Cox, S. M. L., Tippler, M., Castellanos-Ryan, N., Benkelfat, C., Parent, S., … Leyton, M. (2020). Extra-striatal D2/3 receptor availability in youth at risk for addiction. *Neuropsychopharmacology*, *45*(9), 1498–1505. https://doi.org/10.1038/s41386-020-0662-7

Jenkinson, M., Bannister, P., Brady, M., & Smith, S. (2002). Improved optimization for the robust and accurate linear registration and motion correction of brain images. *NeuroImage*, *17*(2), 825–841. https://doi.org/10.1016/s1053-8119(02)91132-8

Kaller, S., Rullmann, M., Patt, M., Becker, G.-A., Luthardt, J., Girbardt, J., … Sabri, O. (2017). Test–retest measurements of dopamine D1-type receptors using simultaneous PET/MRI imaging. *European Journal of Nuclear Medicine and Molecular Imaging*, *44*(6), 1025–1032. https://doi.org/10.1007/s00259-017-3645-0

Kantonen, T., Karjalainen, T., Isojärvi, J., Nuutila, P., Tuisku, J., Rinne, J., … Nummenmaa, L. (2020). Interindividual variability and lateralization of μ-opioid receptors in the human brain. *NeuroImage*, *217*, 116922. https://doi.org/10.1016/j.neuroimage.2020.116922

Kim, M.-J., Lee, J.-H., Juarez Anaya, F., Hong, J., Miller, W., Telu, S., … Innis, R. B. (2020). First-in-human evaluation of [11C]PS13, a novel PET radioligand, to quantify cyclooxygenase-1 in the brain. *European Journal of Nuclear Medicine and Molecular Imaging*, *47*(13), 3143–3151. https://doi.org/10.1007/s00259-020-04855-2

Klein, A., Ghosh, S. S., Bao, F. S., Giard, J., Häme, Y., Stavsky, E., … Keshavan, A. (2017). Mindboggling morphometry of human brains. *PLoS Computational Biology*, *13*(2), e1005350. https://doi.org/10.1371/journal.pcbi.1005350

Lake, B. B., Ai, R., Kaeser, G. E., Salathia, N. S., Yung, Y. C., Liu, R., … Zhang, K. (2016). Neuronal subtypes and diversity revealed by single-nucleus RNA sequencing of the human brain. *Science*, *352*(6293), 1586–1590. https://doi.org/10.1126/science.aaf1204

Lanczos, C. (2006). Evaluation of Noisy Data. *Journal of the Society for Industrial and Applied Mathematics, Series B: Numerical Analysis*. (world). https://doi.org/10.1137/0701007

Larsen, B., Olafsson, V., Calabro, F., Laymon, C., Tervo-Clemmens, B., Campbell, E., … Luna, B. (2020). Maturation of the human striatal dopamine system revealed by PET and quantitative MRI. *Nature Communications*, *11*(1), 846. https://doi.org/10.1038/s41467-020-14693-3

Laurikainen, H., Tuominen, L., Tikka, M., Merisaari, H., Armio, R.-L., Sormunen, E., … METSY group. (2019). Sex difference in brain CB1 receptor availability in man. *NeuroImage*, *184*, 834–842. https://doi.org/10.1016/j.neuroimage.2018.10.013

Lukow, P. B., Martins, D., Veronese, M., Vernon, A. C., McGuire, P., Turkheimer, F. E., & Modinos, G. (2022). Cellular and molecular signatures of in vivo imaging measures of GABAergic neurotransmission in the human brain. *Communications Biology*, *5*(1), 372. https://doi.org/10.1038/s42003-022-03268-1

Marcus, D. S., Harwell, J., Olsen, T., Hodge, M., Glasser, M. F., Prior, F., … Van Essen, D. C. (2011). Informatics and data mining tools and strategies for the human connectome project. *Frontiers in Neuroinformatics*, *5*, 4. https://doi.org/10.3389/fninf.2011.00004

Markello, R. D., Arnatkeviciute, A., Poline, J.-B., Fulcher, B. D., Fornito, A., & Misic, B. (2021). Standardizing workflows in imaging transcriptomics with the abagen toolbox. *eLife*, *10*, e72129. https://doi.org/10.7554/eLife.72129

Mosharov, E. V., Rosenberg, A. M., Monzel, A. S., Osto, C. A., Stiles, L., Rosoklija, G. B., … Picard, M. (2025). A human brain map of mitochondrial respiratory capacity and diversity. *Nature*, *641*(8063), 749–758. https://doi.org/10.1038/s41586-025-08740-6

Naganawa, M., Nabulsi, N., Henry, S., Matuskey, D., Lin, S.-F., Slieker, L., … Huang, Y. (2021). First-in-Human Assessment of 11C-LSN3172176, an M1 Muscarinic Acetylcholine Receptor PET Radiotracer. *Journal of Nuclear Medicine*, *62*(4), 553–560. https://doi.org/10.2967/jnumed.120.246967

Nørgaard, M., Beliveau, V., Ganz, M., Svarer, C., Pinborg, L. H., Keller, S. H., … Knudsen, G. M. (2021). A high-resolution in vivo atlas of the human brain’s benzodiazepine binding site of GABAA receptors. *NeuroImage*, *232*, 117878. https://doi.org/10.1016/j.neuroimage.2021.117878

Patriat, R., Reynolds, R. C., & Birn, R. M. (2017). An improved model of motion-related signal changes in fMRI. *NeuroImage*, *144*(Pt A), 74–82. https://doi.org/10.1016/j.neuroimage.2016.08.051

Power, J. D., Mitra, A., Laumann, T. O., Snyder, A. Z., Schlaggar, B. L., & Petersen, S. E. (2014). Methods to detect, characterize, and remove motion artifact in resting state fMRI. *NeuroImage*, *84*, 320–341. https://doi.org/10.1016/j.neuroimage.2013.08.048

Quackenbush, J. (2002). Microarray data normalization and transformation. *Nature Genetics*, *32 Suppl*, 496–501. https://doi.org/10.1038/ng1032

Radhakrishnan, R., Nabulsi, N., Gaiser, E., Gallezot, J.-D., Henry, S., Planeta, B., … Matuskey, D. (2018). Age-Related Change in 5-HT6 Receptor Availability in Healthy Male Volunteers Measured with 11C-GSK215083 PET. *Journal of Nuclear Medicine*, *59*(9), 1445–1450. https://doi.org/10.2967/jnumed.117.206516

Raffelt, D., Dhollander, T., Tournier, J.-D., Tabbara, R., Smith, R. E., Pierre, E., & Connelly, A. (2017). Bias field correction and intensity normalisation for quantitative analysis of apparent fibre density. *Proceedings of the International Society for Magnetic Resonance in Medicine*, 25, 3541.

Satterthwaite, T. D., Elliott, M. A., Gerraty, R. T., Ruparel, K., Loughead, J., Calkins, M. E., … Wolf, D. H. (2013). An improved framework for confound regression and filtering for control of motion artifact in the preprocessing of resting-state functional connectivity data. *NeuroImage*, *64*, 240–256. https://doi.org/10.1016/j.neuroimage.2012.08.052

Savli, M., Bauer, A., Mitterhauser, M., Ding, Y.-S., Hahn, A., Kroll, T., … Lanzenberger, R. (2012). Normative database of the serotonergic system in healthy subjects using multi-tracer PET. *NeuroImage*, *63*(1), 447–459. https://doi.org/10.1016/j.neuroimage.2012.07.001

Schaefer, A., Kong, R., Gordon, E. M., Laumann, T. O., Zuo, X.-N., Holmes, A. J., … Yeo, B. T. T. (2018). Local-Global Parcellation of the Human Cerebral Cortex from Intrinsic Functional Connectivity MRI. *Cerebral Cortex*, *28*(9), 3095–3114. https://doi.org/10.1093/cercor/bhx179

Shokri-Kojori, E., Naganawa, M., Ramchandani, V. A., Wong, D. F., Wang, G.-J., & Volkow, N. D. (2022). Brain opioid segments and striatal patterns of dopamine release induced by naloxone and morphine. *Human Brain Mapping*, *43*(4), 1419–1430. https://doi.org/10.1002/hbm.25733

Smart, K., Cox, S. M. L., Scala, S. G., Tippler, M., Jaworska, N., Boivin, M., … Leyton, M. (2019). Sex differences in [11C]ABP688 binding: A positron emission tomography study of mGlu5 receptors. *European Journal of Nuclear Medicine and Molecular Imaging*, *46*(5), 1179–1183. https://doi.org/10.1007/s00259-018-4252-4

Tournier, J.-D., Calamante, F., Gadian, D. G., & Connelly, A. (2004). Direct estimation of the fiber orientation density function from diffusion-weighted MRI data using spherical deconvolution. *NeuroImage*, *23*(3), 1176–1185. https://doi.org/10.1016/j.neuroimage.2004.07.037

Tournier, J.-D., Smith, R., Raffelt, D., Tabbara, R., Dhollander, T., Pietsch, M., … Connelly, A. (2019). MRtrix3: A fast, flexible and open software framework for medical image processing and visualisation. *NeuroImage*, *202*, 116137. https://doi.org/10.1016/j.neuroimage.2019.116137

Tournier, J.-D., Yeh, C.-H., Calamante, F., Cho, K.-H., Connelly, A., & Lin, C.-P. (2008). Resolving crossing fibres using constrained spherical deconvolution: Validation using diffusion-weighted imaging phantom data. *NeuroImage*, *42*(2), 617–625. https://doi.org/10.1016/j.neuroimage.2008.05.002

Turtonen, O., Saarinen, A., Nummenmaa, L., Tuominen, L., Tikka, M., Armio, R.-L., … Hietala, J. (2021). Adult Attachment System Links With Brain Mu Opioid Receptor Availability In Vivo. *Biological Psychiatry. Cognitive Neuroscience and Neuroimaging*, *6*(3), 360–369. https://doi.org/10.1016/j.bpsc.2020.10.013

Tustison, N. J., Avants, B. B., Cook, P. A., Zheng, Y., Egan, A., Yushkevich, P. A., & Gee, J. C. (2010). N4ITK: Improved N3 bias correction. *IEEE Transactions on Medical Imaging*, *29*(6), 1310–1320. https://doi.org/10.1109/TMI.2010.2046908

Veraart, J., Novikov, D. S., Christiaens, D., Ades-Aron, B., Sijbers, J., & Fieremans, E. (2016). Denoising of diffusion MRI using random matrix theory. *NeuroImage*, *142*, 394–406. https://doi.org/10.1016/j.neuroimage.2016.08.016

Weathers, F. W., Litz, B. T., Herman, D. S., Huska, J. A., & Keane, T. M. (1993). The PTSD Checklist: Reliability, validity, and diagnostic utility. Paper presented at the annual meeting of the International Society for Traumatic Stress Studies, San Antonio, TX.

Wey, H.-Y., Gilbert, T. M., Zürcher, N. R., She, A., Bhanot, A., Taillon, B. D., … Hooker, J. M. (2016). Insights into neuroepigenetics through human histone deacetylase PET imaging. *Science Translational Medicine*. (world). https://doi.org/10.1126/scitranslmed.aaf7551

Zhang, Y., Brady, M., & Smith, S. (2001). Segmentation of brain MR images through a hidden Markov random field model and the expectation-maximization algorithm. *IEEE Transactions on Medical Imaging*, *20*(1), 45–57. https://doi.org/10.1109/42.906424
